# Polarized cellular mechano-response system for maintaining radial size in developing epithelial tubes

**DOI:** 10.1242/dev.181206

**Published:** 2019-12-02

**Authors:** Tsuyoshi Hirashima, Taiji Adachi

**Affiliations:** 1 Department of Pathology and Biology of Diseases, Graduate School of Medicine, Kyoto University, 6068501, Kyoto, Japan; 2 Institute for Frontier Life and Medical Sciences, Kyoto University, 6068501, Kyoto, Japan

**Keywords:** Epithelial tube, Mechano-response, Quantitative imaging, Tissue polarity, Tissue size control, Vertex model, Mouse

## Abstract

Size control in biological tissues involves multicellular communication via mechanical forces during development. Although fundamental cellular behaviours in response to mechanical stimuli underlie size maintenance during morphogenetic processes, the mechanisms underpinning the cellular mechano-response system that maintains size along an axis of a polarized tissue remain elusive. Here, we show how the diameter of an epithelial tube is maintained during murine epididymal development by combining quantitative imaging, mechanical perturbation and mathematical modelling. We found that epithelial cells counteract compressive forces caused by cell division exclusively along the circumferential axis of the tube to produce polarized contractile forces, eventually leading to an oriented cell rearrangement. Moreover, a mathematical model that includes the polarized mechano-responsive regime explains how the diameter of proliferating tubes is maintained. Our findings pave the way for an improved understanding of the cellular response to mechanical forces that involves collective multicellular behaviours for organizing diverse tissue morphologies.

## Abstract (precis)

**Summary:** Polarized cellular constriction responding to mechanical stress controls the diameter of a developing epithelial tube during murine epididymal development.

## INTRODUCTION

The size of biological tissues is determined by well-coordinated multicellular actions, and its control is essential for various biological processes, including embryonic development, regeneration, and tissue homeostasis. To understand the control of tissue size, it is crucial to not only identify the mechanisms that determine intrinsic tissue size but also ask how the determined size is maintained via multicellular dynamics, although much effort has been made to identify relevant genetic regulators and unravel their chemical interactions at different scales (Penzo-Mendez and Stanger, 2015).

With regard to the maintenance of tissue size, it has been shown in recent years that cell-to-cell communication via mechanical forces is necessary to achieve size maintenance in multicellular systems during development (Brás-Pereira and Moreno, 2018; Eder et al., 2017). An underlying principle of this communication is cellular sensing of and responses to mechanical forces (Guillot and Lecuit, 2013; Petridou et al., 2017). Within a packed tissue, individual cellular events, including cell division and constriction, can be a source of mechanical forces to surrounding cells. The cells perceive mechanical cues through sensor molecules, e.g. mechanosensitive adhesion complexes at cell-cell junctions (Maki et al., 2016; Yonemura et al., 2010), ultimately inducing cytoskeletal activity in response to the perceived mechanical cues. Thus, the cells coordinate their behaviours and actively interact with neighbouring cells by repeated mechano-sensing and activity changes (Ladoux and Mège, 2017). In other words, collective activation of a mechano-response in individual cells is considered to underlie the multicellular actions responsible for maintaining tissue size (Hannezo and Heisenberg, 2019).

Depending on the biological context, tissue size can be viewed from two different perspectives: the volume of a tissue during tissue homeostasis and the length along a tissue axis during tissue development. For each of these perspectives, previous studies have investigated the maintenance of tissue size from the viewpoint of the cellular mechano-response at a multicellular scale. For the maintenance of tissue volume, for example, cell proliferation is counterbalanced by cell death or extrusion from the tissue, and these coordinated cellular behaviours are invoked in response to cell-crowding pressure generated by proliferating cells (Eisenhoffer et al., 2012; Gudipaty et al., 2017; Hufnagel et al., 2007; Shraiman, 2005). This system is a highly coordinated regulation system that promotes homeostasis of tissue volume. On the other hand, for the maintenance of tissue length, such as the maintenance of the cross-sectional diameter of proliferating epithelial tubes (Andrew and Ewald, 2010), the cellular mechano-response should work anisotropically to promote tube elongation, as a net increase in the tissue volume should be distributed along the longitudinal axis but not the circumferential axis.

The maintenance of length in developing tissues can be considered another aspect of anisotropic tissue growth, which gives rise to a variety of tissue shapes. Recent studies have revealed that two main factors associated with cellular behaviours underpin anisotropic tissue growth. One factor is cell division orientation, which is mainly determined by the mitotic spindle orientation during cytokinesis. It has been shown that the orientation of cell division in some cases directly controls the shape of growing tissues, and increasing evidence indicates that the misorientation of cell division leads to abnormal tissue morphology, as demonstrated in various organs and species (Fischer et al., 2006; Morin and Bellaïche, 2011; Tang et al., 2011, 2018). The other factor is cell intercalation, which is active cellular rearrangement that involves changing the position of cell junctions between neighbouring cells (Guillot and Lecuit, 2013; Walck-Shannon and Hardin, 2014). It has been clearly demonstrated that cell intercalation is driven by polarized non-muscle myosin II localization to cell-cell junctions, and the cumulative effect of local tissue dynamics results in large anisotropic deformation at the tissue scale (Bertet et al., 2004; Nishimura et al., 2012; Zallen and Blankenship, 2008). These cellular events generate mechanical forces and thereby impact surrounding cells in multicellular packed tissues; however, it remains unclear how they collectively work to maintain tissue length in developing tissues.

Here, we utilized epididymal tubes in murine embryos as an experimental model system in which to study the maintenance of tube radial size during development. The epididymal tube is a single mono-layered epithelial tube that exhibits dynamic morphological changes, including bending and folding, from embryonic day (E) 15.5 (Hirashima, 2014, 2016; Joseph et al., 2009). Regarding epididymal morphogenesis, Xu et al. have shown that circumferentially oriented cell intercalation driven by actomyosin constriction is involved in the control of tube diameter (Xu et al., 2016). They showed that the disruption of oriented cell intercalation increases the diameter of epididymal tubes while shortening the longitudinal length. Supposing that oriented cell intercalation is constitutively sustained in a developing epididymal tube, the tube diameter would become gradually smaller during development. Interestingly, however, the diameter of the developing epididymal tube is almost constant even though the tissue volume increases as a result of incessant cell proliferation in the tubes (Hirashima, 2014; Tomaszewski et al., 2007). Therefore, the study by Xu et al. (2016) motivated us to identify the unknown regulatory system that maintains the diameter of epididymal tubes during development. We employed an interdisciplinary approach by combining quantitative imaging, mechanical perturbation and mathematical modelling and found that epididymal cells counteract mechanical forces exclusively along the circumferential axis of the tube. We propose that a polarized mechano-response system operates at a supra-cellular scale to maintain tube diameter at the whole-tissue scale.

## RESULTS

### Cell division orientation is unbiased within the monolayer of developing epididymal tubes

**Fig. 1.**
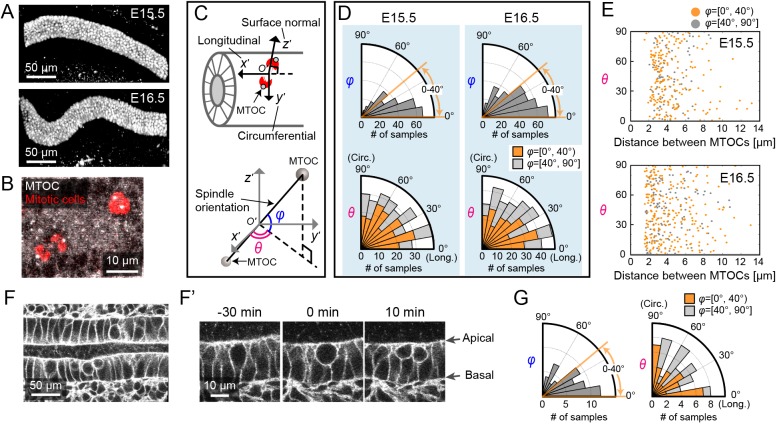
**Quantitative 3D analysis of cell division orientation in epididymal tubes.** (A) Immunofluorescence images of Pax2 at E15.5 and E16.5. (B) Maximum intensity projection of immunofluorescence image for pHH3 (mitotic cells, red) and γ-tubulin (MTOC, white). (C) Local polar coordinate system (φ, θ) for the measurement of spindle or cell division orientation in mitotic cells in the tube monolayer. (D) Angle distributions (φ and θ) of the spindle orientation. Colours in the θ distribution represent samples for which φ ranges from 0-40° (orange, *n*=197 at E15.5 and *n*=240 at E16.5) and from 40-90° (grey, *n*=80 at E15.5 and *n*=118 at E16.5). The samples were collected from six embryos. (E) Scatter plots of the distance between MTOCs and the angle θ at E15.5 and E16.5. (F) Snapshot of time-lapse images of the cell membranes of developing epididymal tubes in reporter mice. (F′) Magnified images of F, focusing on cytokinesis within the epithelial monolayer. The initial time point was immediately before cytokinesis. (G) Angle distributions (φ and θ) of the cell division orientation from the live-imaging data. Colours in the θ distribution represent samples for which φ ranges from 0-40° (orange, *n*=39) and from 40-90° (grey, *n*=17). The samples were collected from four embryos.

Because cell division orientation is known as a key determinant for the morphogenesis of developing tubes (Fischer et al., 2006; Tang et al., 2011), we examined cell division orientation in mouse epididymal tubes at E15.5 and E16.5. As the tube diameter is unchanged from E15.5 to E16.5 (Fig. 1A) whereas its longitudinal length increases by 1.54-fold (Hirashima, 2014), the cell division orientation was thought to be biased parallel to the longitudinal direction of the tube. We first performed immunostaining for phospho-histone H3 (pHH3) and γ-tubulin as markers of mitotic cells and microtubule organizing centres (MTOCs) (Fig. 1B), and obtained distributions for the two angles of the spindle orientation, radial orientation (φ) and longitudinal orientation (θ), without destroying the 3D tissue structure (Fig. 1C, Fig. S1, Materials and Methods). The distribution of φ showed that almost 70% of the measured spindles are within the range of 0-40°, indicating that the majority of the spindle orientations are parallel to the epithelial layer (Rayleigh test, *P*<0.001 at E15.5 and E16.5). On the other hand, the distribution of θ showed that the spindle orientation is not biased to either the longitudinal or circumferential axis (Rayleigh test, *P*≥0.05 at E15.5 and E16.5) (Fig. 1D). Furthermore, there was no correlation between the angle θ and the distance between MTOCs (*r*=−0.07 at E15.5, *r*=−0.02 at E16.5, where *r* is Pearson's correlation coefficient) (Fig. 1E); if the spindles were collectively directed to a specific orientation, the relationship between the angle and the distance would exhibit a trend because the distance between MTOCs becomes longer as cell cycle progresses in M phase. Our result suggests that spindle orientation might not progress to a specific orientation in the later stage of mitosis, unlike lung development (Li et al., 2018).

To investigate the cell division orientation in developing tubes, we dissected the epididymis at E15.5 and performed live-imaging analysis of epididymal tube cells using *ex vivo* organ culture systems. To visualize the cell membrane, we crossed the R26R-Lyn-Venus line (Abe et al., 2011) and the Pax2-Cre line (Ohyama and Groves, 2004) to create a conditional fluorescence reporter line. Because the epithelial tubes are located more than 100 µm away from the capsule of the epididymis, we used a multiphoton excitation microscope for deep-tissue live imaging in explant cultures (Fig. 1F). From live imaging, we found that the epithelial cells moved to the apical side of the epithelial layer, followed by rounding and cytokinesis (Fig. 1F′). In addition, the cells remained within the epithelial layer, in contrast to what is observed in the mouse ureteric bud during development (Packard et al., 2013). This observation implies that the mitotic cells physically interact with neighbouring cells on the apical side of the epithelial layer and transmit pushing forces directly to their neighbours, contributing to morphological changes of the tubes. Then, we examined the two angles (φ and θ) of the cell division orientation from the live-imaging data and found that their distributions were similar to those of spindle orientation (Fig. 1D,G). The major fraction of the cell divisions in φ falls into the range of 0-40° (70%), indicating that cell division occurs mostly parallel to the epithelial layer (Rayleigh test, *P*<0.001). Moreover, the distribution of θ is not biased to either the longitudinal or circumferential axis of the tubes (Rayleigh test, *P*≥0.05). These results clearly indicate that the developing epididymal tubes do indeed include cell division along the circumferential axis of the tube, which is supposed to result in an increase in tube diameter. This finding led us to question how epithelial cells could actively counteract circumferential cell division to maintain tube diameter.

### Actomyosin constriction is polarized along the circumferential axis of tubes

**Fig. 2.**
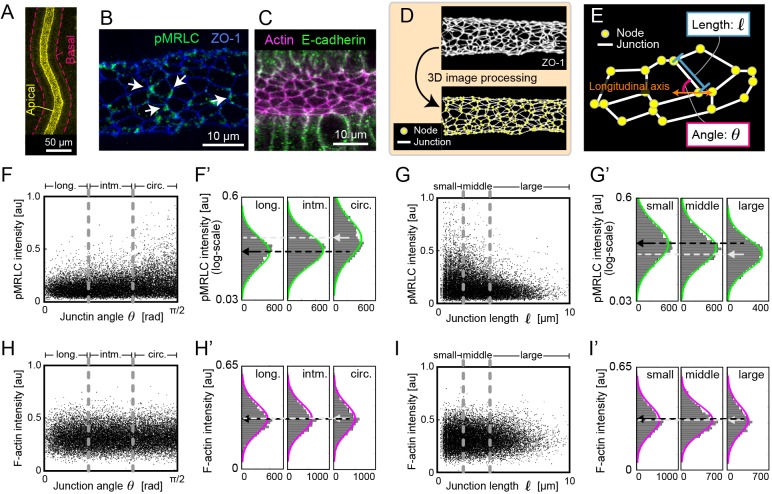
**Quantitative analysis of the active myosin distribution in epididymal tubes.** (A) Immunofluorescence image of pMRLC (yellow). The basal sides of the epididymal tubes are marked by magenta dashed lines. (B) Immunofluorescence image of pMRLC (green) and ZO-1 (blue). Arrows indicate pMRLC localization to circumferential apical junctions. (C) F-actin staining (magenta) and immunofluorescence of E-cadherin (green) on the apical side of epididymal tubes. (D) Digital processing of the ZO-1-stained images detects nodes and junctions of apical surfaces. (E) Illustration of the junction length *l* and the junction angle θ, measuring the angle from the longitudinal axis of the tubules. (F-G′) Relationship between pMRLC intensity and the junction angle/length. The samples were categorized into three groups (F,G, dashed lines) and summarized as histograms on a logarithmic scale (F′,G′). Black arrows represent the mean intensity in the longitudinal (long.)/small group, and grey arrows represent the mean intensity in the circumferential (circ.)/large group. *n*>5000 from 8 embryos. (H-I′) Relationship between F-actin intensity and the junction angle/length. The samples were categorized into three groups (H,I, dashed lines) and summarized as histograms on a linear scale (H′,I′). Black and grey arrows represent the parameters described for F′ and G′. *n*>5000 from 8 embryos.

To examine the mechanical factors that prevent circumferential cell division, we focused on phosphorylated myosin regulatory light chain (pMRLC), a marker of active tension generated via actomyosin constriction in non-muscle cells. We first examined the localization of pMRLC in the epididymis at E16.5 by whole-tissue immunofluorescence and found that pMRLC localizes only to the apical side of epithelial tubes (Fig. 2A). To quantify the spatial distribution, we performed fluorescence staining for active tension generators, including pMRLC and F-actin, with staining for the apical tight junction marker zonula occludens-1 (ZO-1) at E15.5 and E16.5. From this experiment, we found that pMRLC localizes to a portion of the apical cellular junctions, whereas F-actin accumulates uniformly at all apical junctions (Fig. 2B,C).

Using the ZO-1 signal, we measured the mean intensity of pMRLC at junctions, the junction angle from the tube longitudinal axis θ, and the junction length *l* through automatic extraction for each apical cell junction (Fig. 2D,E, Fig. S2A; 96% of the whole extracted edges were evaluated; see Materials and Methods). For evaluation, we categorized the junction angle into three groups: longitudinal (long.), 0≤θ≤30; intermediate (intm.), 30<θ<60; and circumferential (circ.), 60≤θ≤90 (Fig. 2F,F′). The histograms for each group show that the pMRLC distribution in the circumferential group was higher than that in the longitudinal and intermediate groups, which is significant compared with a ZO-1 profile (Fig. 2F′, Fig. S2B) (one-way ANOVA, *P*<0.001). This indicates that active myosin at apical junctions is polarized along the circumferential axis of the tubes. As for the junction angle, we also categorized the junction length into three groups: small, 0≤*l*≤2 μm; middle, 2<*l*<4 µm; and large, 4≤*l*≤10 µm (Fig. 2G,G′). We found that the pMRLC distribution in the large group was lower than that in the small and middle groups, indicating that active myosin tended to localize more at shorter junctions (one-way ANOVA, *P*<0.001) (Fig. 2G′, Fig. S2C). In addition to the pMRLC quantification, we performed the same analysis for F-actin and found that there was no trend between the F-actin intensity and the junction angle or between the intensity and the junction length (Fig. 2H-I′) (one-way ANOVA, *P*≥0.05). These quantifications revealed that active myosin, but not F-actin, is polarized at apical junctions along the circumferential axis of developing epididymal tubes.

### Cell division triggers polarized myosin activation to maintain the tube diameter

**Fig. 3.**
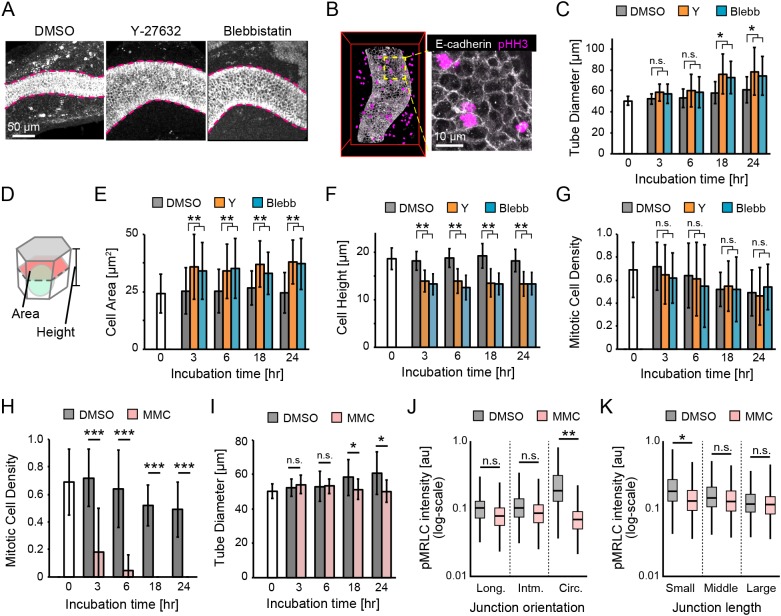
**Pharmacological assay of myosin activity and cell division.** (A) Immunofluorescence images of E-cadherin in the epididymis after 1 day of Y-27632 (10 µM) or blebbistatin (10 µM) treatment. The dashed lines represent the periphery of the tubes. (B) Co-immunostaining for E-cadherin and pHH3 after blebbistatin treatment. (C) Tube diameter with Y-27632 (Y) or blebbistatin (Blebb) throughout the study period. *n*=5. (D) Schematic of the measurement of cell area and height. (E-G) Cell area, cell height and mitotic cell density with Y-27632 or blebbistatin throughout the study period. *n*>100 from 5 embryos for the cell area, *n*>30 from 5 embryos for the cell height, and *n*=5 for the mitotic cell density. (H,I) Time course of mitotic cell density and tube diameter with mitomycin C (MMC) throughout the study period. *n*=5. (J) pMRLC intensity with different apical junction orientation: longitudinal, intermediate and circumferential. *n*>2000 from 5 embryos. (K) pMRLC intensity with different apical junction length: small, medium and large. *n*>2000 from 5 embryos.

We then examined the impact of actomyosin constriction and cell division on the tube diameter in a pharmacological assay. First, we applied Y-27632 (10 µM) to inhibit Rho-associated protein kinase (ROCK), an upstream regulator of myosin activity, or blebbistatin (10 µM) to inhibit actomyosin constriction by non-muscle myosin II in epididymis samples dissected at E15.5 in explant cultures. At 1 day after inhibitor treatment with either compound, the tubes were remarkably bloated, indicating that ROCK-dependent actomyosin constriction at apical junctions is required for the maintenance of tube diameter (Fig. 3A). Moreover, we found that the cells transition to M phase of the cell cycle even with inhibitor treatment (Fig. 3B). We measured the tube diameter after various incubation times with the inhibitors and found by 18 h after inhibitor treatment the tube diameter was ∼1.4-fold larger compared with that of the control, whereas its growth rate increased by ∼1.1-fold at most in 6 h (Mann–Whitney *U*-test, *P*<0.05) (Fig. 3C; Materials and Methods). For further analysis, we quantified cell area, cell height, and the number of mitotic cells after incubation with the inhibitors (Fig. 3D-G). The cell area was measured at the middle along the apico-basal axis, and the cell height was measured as the apico-basal length (Fig. 3D). The cell area increased by ∼1.4-fold and the cell height decreased to ∼70% after treatment for 3 h, and these changes were retained afterwards (Mann–Whitney *U*-test, *P*<0.01) (Fig. 3E,F). These results indicate that the inhibitors altered the cell shape without changing the cell volume within 3 h (Fig. S3); thus, the sum of the cell shape changes reflects the change in tube diameter during the early phase of inhibitor treatment (Fig. 3C). Regarding the effects on cell division, the number of mitotic cells in the tubes was almost the same as that of the control (Mann–Whitney *U*-test, *P*≥0.05) even after inhibitor treatment, although this parameter decreased with increasing incubation time in all cases (Fig. 3G), indicating that, at the concentrations used, the inhibitors do not affect the control of cell division. These results indicate that the endogenous myosin activity in the tubes contributes to suppression of the tube radial expansion caused by cell division in epididymal tubes.

Next, we examined whether cell division in the tubes affects the tube diameter and myosin activity. We evaluated whether cell cycle entry into M phase in the developing epididymal tubes was inhibited by mitomycin C (MMC) treatment at a concentration of 3 µM in explant cultures and found that the cell division was completely inhibited by 18 h after the treatment (Fig. 3H). The tubes subjected to MMC treatment maintained their diameter during the culture, but they expanded slightly at 18 h in the control (Mann–Whitney *U*-test, *P*<0.05) (Fig. 3I), indicating that cell division is required for tube radial expansion. Regarding the effects of myosin activity, we measured pMRLC intensity as myosin activity at apical junctions by whole-tissue immunofluorescence and found that MMC treatment led to a significant decrease in pMRLC intensity at the circumferential apical edges compared with that of the control after 18 h of treatment, meaning that the myosin anisotropic localization is lost as a result of MMC treatment (Mann–Whitney *U*-test, *P*<0.01) (Fig. 3J,K). This result indicates that cell division might induce the activation of polarized myosin along the circumferential axis of tubes.

It has been reported that polarized pMRLC localization drives cell intercalation mediated by junction shrinkage in the developing epididymis (Xu et al., 2016), as demonstrated in other tissues (Bertet et al., 2004; Lienkamp et al., 2012; Nishimura et al., 2012; Shindo and Wallingford, 2014; Yamamoto et al., 2009). Hence, the polarized localization of pMRLC to circumferential junctions drives oriented cell intercalation, eventually leading to radial shrinkage and longitudinal elongation of the tube. In contrast, cell division directed along the circumferential axis within the tube monolayer results in tube radial expansion (Tang et al., 2018). Therefore, maintaining tube diameter requires an appropriate balance between polarized myosin activation, which drives cell intercalation, and circumferential cell division. Overall, it is reasonable to consider that circumferential cell division triggers polarized myosin activation, which drives cell intercalation, but not vice versa, to achieve the balance required for maintenance of the tube diameter.

### Cell division attenuates the mechanical tension in neighbouring cells

Cell division is known to have a mechanical influence on surrounding cells in epithelial tissues owing to its outward rounding pressure (Stewart et al., 2011; Tang et al., 2018). Therefore, we examined the mechanical impacts of cell division in the epithelial tube using computer simulations of a mechanics-based model. Here, we employed the vertex dynamics model to represent multicellular dynamics (Fletcher et al., 2014; Kursawe et al., 2015; Nagai and Honda, 2001; Rauzi et al., 2008). The apical side of the tube cells was modelled as a flexible multicellular membrane in three-dimensional space according to earlier studies (Du et al., 2014; Osterfield et al., 2013) because force sensation and generation are concentrated at the apical junctions (see Materials and Methods for more details). To evaluate mechanical stress in the tube cells, we introduced a scalar quantity, i.e. cell tension anisotropy, ρ_α_. ρ_α_ increases when the cell tension along the circumferential axis becomes weaker than the cell tension along the longitudinal axis (see Materials and Methods).

**Fig. 4.**
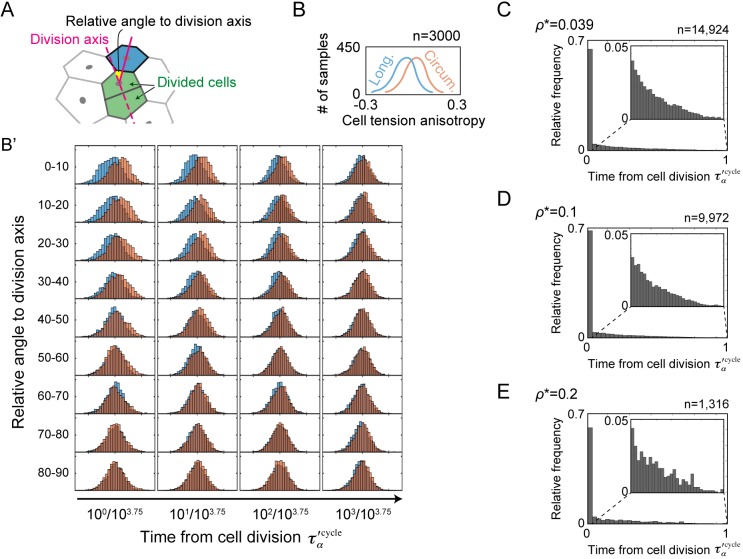
**Computational analysis of cell stress influenced by cell division in tubes.** (A) The cell tension anisotropy was calculated in neighbouring cells (e.g. blue) to the divided cells (green). (B) Explanatory illustration for the histograms of cell tension anisotropy against the cell division. Orange indicates the case of circumferential cell division and blue indicates the case of longitudinal cell division. (B′) Histograms of cell tension anisotropy in various cases of the relative angle to the cell division axis (*y*-axis) compared with the time from cell division τ′_α_^cycle^ of neighbouring cells (*x*-axis). *n*=3000. (C-E) Histograms of the elapsed time after the cell division τ′_α_^cycle^ when the cell tension anisotropy is over various thresholds, i.e. ρ*=0.039, 0.1 and 0.2, in neighbouring cells on the circumferential axis. The tail of histograms is magnified in the insets. *n*=14,924 for ρ*=0.039, *n*=9972 for ρ*=0.1, and *n*=1316 for ρ*=0.2.

We first examined the cell tension anisotropy of neighbouring cells to divided cells. In particular, we focused on the dependency against relative position to the cell division axis (Fig. 4A) and elapsed time from the cell division. Histograms of the cell tension anisotropy were drawn for the case of longitudinal cell division and that of circumferential cell division (Fig. 4B,B′). The results indicated that cell division mechanically affects cell tension anisotropy in the neighbouring cells of divided cells on the division axis. This is intuitive because the divided cells push the neighbouring cells along the division axis. Thus, the tension in the neighbouring cells along the axis is attenuated. Furthermore, we found that the tension attenuation due to cell division gets smaller over time after cell division.

Is cell division the only source of tension attenuation in cells? To answer this question, we next plotted histograms of the time after cell division of cells on the circumferential cell division axis when the cell tension anisotropy ρ_α_ is over various thresholds: ρ*=0.039, 1 and 0.2 (Fig. 4C-E; ρ*=0.039 is the mean of cell tension anisotropy in the upper left case of Fig. 4B′). Although most of the cells are in the stage that comes immediately after the cell division, the histograms show the long-tailed distribution of the cell stages at any given thresholds.

These results led to two conclusions. First, most of the cells that receive significant mechanical impact from the cell division are positioned on the cell division axis soon after the cell division. Second, cell tension can be varied independently of cell division, rationalizing the mechano-responsive regime presented below.

### Cell intercalation tends to be triggered by circumferential cell division

**Fig. 5.**
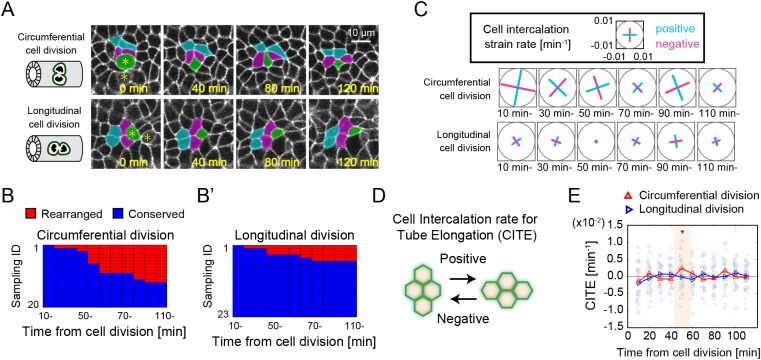
**Live-imaging analysis of local cell behaviours around dividing cells.** (A) Snapshots from live-imaging data in the case of circumferential cell division (upper) and longitudinal cell division (bottom). The initial time point was the time of cytokinesis completion. Asterisks represent daughters of divided cells, one of which is coloured artificially in green. Some cells neighbouring the daughter cell are also coloured for easy visualization. (B,B′) Chart of the T1 process in each sample for circumferential cell division (B) and longitudinal cell division (B′). Cells that preserved their relations with neighbouring cells after cell division are represented in blue, and those that experienced the T1 process (rearrangement) are represented in red. *n*=20 for circumferential cell division and *n*=23 for longitudinal cell division. The samples were collected from four embryos. (C) Cell intercalation strain rate of the time-lapse images shown in A in the case of circumferential cell division and longitudinal cell division. The scale and sign for the cell intercalation strain rate are shown in the upper box. (D) Schematic of the cell intercalation rate in the context of tube elongation (CITE). (E) The time series of CITE for the cases of circumferential cell division (red, *n*=20) and longitudinal division (blue, *n*=23). The highlighted time period indicates the period during which the mean value for circumferential cell division was not statistically zero.

During the process of cell mitosis, the neighbouring cells are pushed and deformed on the apical side of the epithelial layer by the mitotic cells, as shown in Fig. 1E′, and those cells are subjected to mechanical forces due to cell division as suggested in the previous section. To investigate whether cell intercalation is triggered by circumferential cell division, we used reporter mice to mark the cell membrane of epididymal tubes and analysed the live-imaging data in explant cultures dissected at E15.5. According to the model suggestions, we focused on cells that were both adjacent to the mitotic cells and located on an extension of the mitotic cell division axis (Fig. 5A, Movie 1).

We then evaluated cell behaviours around the mitotic cells according to the two different categories of cell division orientation: circumferential cell division (0≤θ≤30) and longitudinal cell division (60≤θ≤90). Careful observation led to the discovery of characteristic behaviours in those cells: the topological rearrangement (T1) process of cellular junctions after cell division occurred more frequently in circumferential cell division than in longitudinal cell division. The T1 process occurred at a rate of 60% (*n*=12/20 from 4 different embryos) for circumferential cell division, whereas it occurred at a rate of 22% (*n*=5/23 from 4 different embryos) for longitudinal cell division within 2 h of cell division (Fig. 5B,B′). For further quantification of oriented cell intercalation behaviour, we measured the cell intercalation strain rate (Blanchard et al., 2009) for circumferential or longitudinal cell division (Fig. 5C, Fig. S4; see Materials and Methods for the definition) and calculated the cell intercalation rate for tube elongation (CITE) as a measure of oriented cell intercalation for elongating the tubes. Positive/negative values for the CITE indicated that the cell intercalations underwent tube elongation/shortening, respectively (Fig. 5D; Materials and Methods). From the quantitative analysis, we found that oriented cell intercalation tended to occur approximately 50 min after circumferential cell division (*t*-test *P*<0.05 in the time range: 50-60 min in the case of circumferential cell division) (Fig. 5E).

Regarding the time scale of cellular behaviours as an output response to mechanical cues, oriented cell intercalation should be accomplished through rapid biochemical processes, such as phosphorylation. Taken together, these results suggested that circumferential myosin activity would be enhanced in response to mechanical stimuli provided to the tube cells by neighbouring cells, ultimately driving oriented cell intercalation.

### Polarized myosin activity is enhanced by circumferential compression of epididymal tubes

**Fig. 6.**
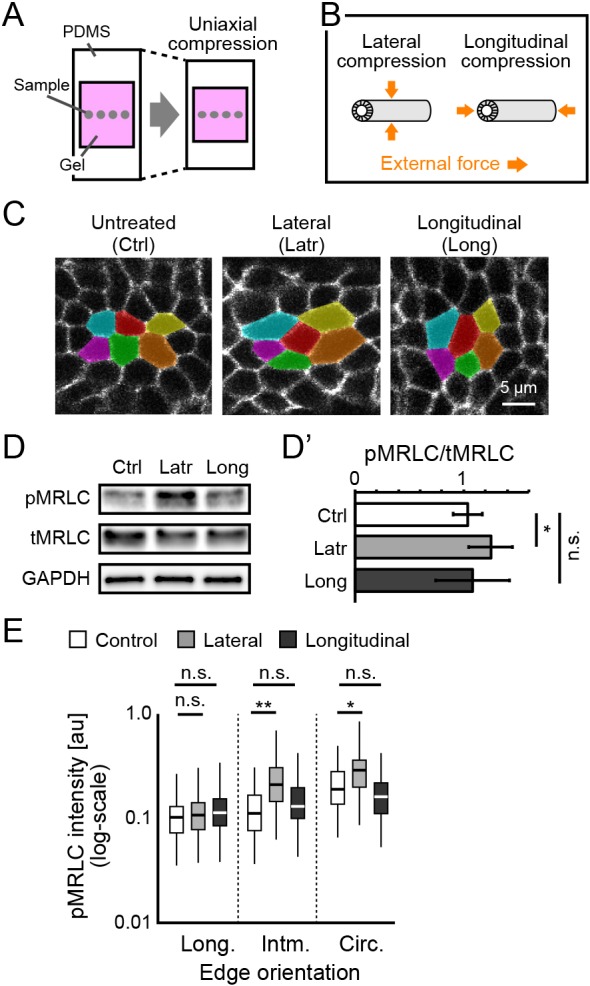
**Mechanical perturbations reveal an anisotropic cellular response in myosin activity.** (A,B) Schematics of the uniaxial compression assays. (C) Morphology of the tubule cells following mechanical compression. The cells were manually coloured for visualization of cell shape. (D,D′) Immunoblotting and quantification of myosin activity for the mechanical treatments. The data are normalized to the mean value in the control. *n*=9. (E) pMRLC intensity in the apical edge orientation of the tubes after mechanical treatments. *n*>4000 from 9 embryos.

To verify our hypothesis, we applied uniaxial compressive strain (∼33%) to isolated epididymis embedded in a hydrogel using a flexible polydimethylsiloxane (PDMS) chamber and evaluated the myosin activity as a cellular response to compression (Fig. 6A). We performed the compression assay for three groups – non-compression as the control, lateral compression and longitudinal compression – to examine the cellular responses to different mechanical perturbations (Fig. 6B).

After 10 min of continuous compression, we quantified the cell shape changes caused by uniaxial compression. For quantification, we extracted the shape of tube cells from immunofluorescence images of E-cadherin (also known as cadherin 1) (Fig. 6C) and measured the aspect ratio and the major axis angle from best-fit ellipses of the cell shapes. The shape of cells subjected to uniaxial compression was significantly different from that of cells in the non-compression group, confirming that uniaxial compressive strain could apply force to the epididymal tube cells along the compressive axis (Fig. S5A-C).

We then measured the level of pMRLC 20 min after continuous compression to verify whether anisotropic mechanical compression alters myosin activity. Compression did not influence the number of cells undergoing mitosis (Kruskal–Wallis test, *P*≥0.05) (Fig. S5D). We quantified the myosin activity as the relative intensity of pMRLC to total MRLC (tMRLC) by western blotting and found that myosin activity was stronger in the lateral compression group than in the other groups. This result indicates that the myosin activity is exclusively enhanced by lateral compression (Friedman test, *P*<0.05) (Fig. 6D,D′). Furthermore, we quantified myosin activity at apical junctions by whole-tissue immunofluorescence and found that lateral compression enhanced myosin activation at circumferential apical junctions compared with that of the control, but this change was not observed at longitudinal apical junctions (Kruskal–Wallis test, *P*<0.05) (Fig. 6E). For longitudinal compression, myosin activity was not significantly different in any orientation for apical junctions compared with the control case (Kruskal–Wallis test, *P*≥0.05) (Fig. 6E). These results suggest that epididymal tube cells possess a system in which MRLC phosphorylation is triggered in response to compression, specifically along the circumferential axis of the tube.

### An anisotropic mechano-response system maintains the diameter of developing tubes

**Fig. 7.**
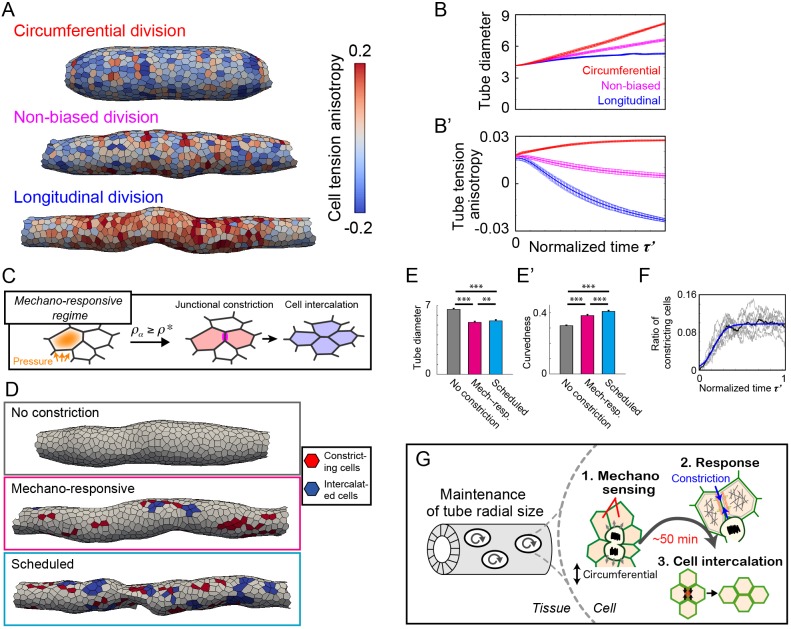
**Mathematical model including anisotropic cellular mechano-responses for maintaining tubule radial size.** (A) The vertex model simulation of proliferating tubes in different cell division orientations. The colour represents the cell tension anisotropy. (B,B′) Time course of morphological and mechanical quantities along the normalized simulation time τ′. See Materials and Methods for those quantities and the definition of τ′. *n*=15. (C) Schematic of the mechano-responsive regime. In this regime, circumferential junction constriction starts when the cell tension anisotropy becomes larger than the threshold ρ* due to the pressure from circumferentially neighbouring cells. (D) Tubes generated in different regimes: no constriction, mechano-responsive, and scheduled. The red regions represent cells constricting the circumferential junction, and blue represents cells that have completed cell intercalation. (E,E′) Resulting morphological quantities of tubes, tube diameter and tube curvedness, in different regimes. *n*=10. (F) The ratio of constricting cells to total cells over the normalized simulation time τ′ in the mechano-responsive regime. Raw data from simulations are shown in grey (*n*=10), the mean value in black and model fitting in blue. (G) Model for the maintenance of tubule radial size in the developing epididymis. Epididymal cells have ability to counteract compressive forces exclusively along the circumferential axis of the tube to induce oriented cell rearrangement via production of polarized contractile forces.

Finally, we investigated how mechano-responsive cellular behaviours impact tube morphology using the vertex model (see Materials and Methods). First, we examined the effects of tube shape and its mechanical state on cell division in the absence of active constriction via myosin regulation. In the simulation, we confirmed that the virtual epithelial tubes grew along the division orientation, indicating that cell division along the circumferential axis is attributed to continuous radial growth (Fig. 7A,B, Fig. S6, Movie 2). We also found that the cell division orientation affects mechanical stress in each cell in the virtual tubes. As shown in Fig. 7A,B′, the cell tension anisotropy in the tubes depends on the cell division orientation and corresponds to the growth direction of the tubes. This analysis shows that circumferential cell division entails an increase in cell tension anisotropy locally in the tissue, resulting in radial tube expansion.

Next, we examined the changes in the tube diameter in a mechano-responsive regime that corresponded to our hypothesis and in an alternative regimes. We implemented the mechano-responsive regime in the mathematical model as follows: the cells keep constricting one of the circumferential junctions for a parameterized period as a result of the cell tension anisotropy ρ_α_ reaching the threshold value ρ* due to the reception of intense pressure by neighbouring cells (Fig. 7C, Fig. S7A-B′, Materials and Methods). The increase in cell tension anisotropy is not necessarily caused by the pushing force of cell division in neighbouring cells, but is instead due to other factors, including the accumulation of local tissue stress in an irregular cell topology in the proliferating epithelial tissue (Fig. 4B′). Thus, we rationalized the introduction of a mechano-responsive regime into our model instead of considering only cell division. The numerical investigation clearly suggests that, compared with the case of no regulation, the mechano-responsive regime can support spatiotemporally constant tubes with smaller radial sizes (Fig. 7D-E′, Movie 3).

We also performed simulations in a counterpart regime, the ‘scheduled’ regime, in which the timing for junction constriction was predetermined and the constricting cells were randomly selected. To determine the schedule of constriction, we sampled the ratio of constricting cells along the normalized simulation time in the mechano-responsive regime and obtained the function by fitting the mean curve data (Fig. 7F, Materials and Methods). In the scheduled regime, the tubes could form with irregular surfaces and non-constant radial size along the longitudinal axis, but the radial size was smaller than that observed for no regulation or for the mechano-responsive regime (Fig. 7D-E′).

In the scheduled regime, the oriented cell intercalation that leads to the local shrinkage of tubes occurs independently of variations in cell tension, resulting in tubes with an irregular surface. In the mechano-responsive regime, however, mechanical stress at the supra-cellular scale can be modulated due to the active multicellular movement that immediately responds to mechanical perturbations. Therefore, the smooth curved surface throughout the longitudinal axis of the tubes can be realized in this regime. These results indicate that the mechano-responsive regime, as an embodiment of our hypothesis, explains the maintenance of diameter in developing tubes (Fig. 7G).

## DISCUSSION

Using a combination of quantitative imaging and mechanical perturbation assays, we found that epididymal cells trigger polarized myosin activation by responding to mechanical forces exclusively in the circumferential axis of the tube. This finding indicates that cells actively generate contractile forces in response to the compressive force caused by the behaviour of other constitutive cells during epididymal development. Moreover, the cells should have an intrinsic system for sensing mechanical force exclusively in the circumferential axis of the epididymal tubes. Finally, we demonstrated that a mathematical model that includes the mechano-responsive regime could reproduce the maintenance of tube diameter, consistent with observations during mouse epididymal development. Thus, we propose a system in which the polarized mechano-responsive behaviour of cells at the supra-cellular scale organizes the maintenance of the tube diameter at the whole-tissue scale (Fig. 7G).

Here, we propose a multicellular system composed solely of epithelial cells in the monolayer tube; however, the mechanisms for regulating the tube diameter are multiple and depend on the embryonic stage. Our results clearly show that no pMRLC localization can be found in the mesenchyme of the epididymis from E15.5 to E16.5, indicating that the actomyosin-dependent active constriction from the surrounding mesenchyme to the epithelial tubes is subtle at this stage. However, mesenchymal cells surrounding the epididymal tubes differentiate into smooth muscle progenitors from E16.5 during development and gradually gain the potential to generate contractile forces radially to the tubes (Hirashima, 2014; Robaire et al., 2006). In addition to epididymal development, it has also been reported that the radial contraction of smooth muscle cells is effective for determining tube radial morphology in the vertebrate intestine, lung and trachea (Hirashima and Adachi, 2015; Kishimoto et al., 2018; Shyer et al., 2013). Therefore, it is reasonable to consider that external contractile forces caused by mesenchymal differentiation contribute to suppression of the radial enlargement of epididymal tubes, as well as the proposed mechanism during the late stage of epididymal morphogenesis.

In addition to size homeostasis, the polarized cellular mechano-response can be considered as a core regulatory mechanism for tube morphogenesis during development. In general, the anisotropic growth of proliferating epithelial tubes causes the generation of compressive forces, eventually leading to the buckling-induced pattern formations that have been reported in various organs, including the fly trachea and the chick midgut (Dong et al., 2014; Hirashima, 2014; Savin et al., 2011). Thus, the proposed polarized mechano-response system might work as a regulatory principle that underlies mechanisms of self-organized tissue morphogenesis.

To understand this system further, future work will need to identify the molecular machinery that senses anisotropic mechanical force in tissues and determine how the received mechanical stimuli are chemically transduced to activate myosin at apical junctions. It is tempting to speculate that the directional preference of cells would be provided by regulators of planar cell polarity (PCP) that promote downstream myosin phosphorylation (Bosveld et al., 2012; Guillot and Lecuit, 2013; Nishimura et al., 2012), such as Vang-like (VANGL) and tyrosine-protein kinase-like 7 (PTK7). These proteins are well aligned circumferentially along the junctions of epididymal tube cells, and loss of PCP disrupts actomyosin-mediated cell intercalation, resulting in radial tube expansion (Xu et al., 2016) (Fig. S7C-E′) and renal tube development (Karner et al., 2009; Kunimoto et al., 2017). Another possibility is that actin filaments may function as polarity tension sensors in cooperation with unknown molecules that specify the circumferential axis because those filaments have been reported to sense tension reduction (Galkin et al., 2012; Hayakawa et al., 2011). We believe that our findings will advance our understanding of the structural components and dynamic properties of the polarized mechano-response system that controls tissue morphogenesis.

## MATERIALS AND METHODS

### Experiments

#### Animals

For live-imaging analysis, we used fluorescence reporter mice produced by crossing the Pax2-Cre line (Ohyama and Groves, 2004) (a gift from T. Furukawa, Osaka University, Japan) and R26R-Lyn-Venus line (Abe et al., 2011). Otherwise, we used imprinting control region (ICR) mice purchased from Japan SLC. We designated the midnight preceding the plug as E0.0, and all mice were sacrificed by cervical dislocation to minimize suffering at E15.5 or E16.5. All the animal experiments were approved by the local Ethical Committee for Animal Experimentation of Institute for Frontier Life and Medical Sciences, Kyoto University, and were performed in compliance with the Guide for the Care and Use of Laboratory Animals at Kyoto University.

#### Antibodies and fixative solutions for immunostaining

We used the following primary antibodies at 1:200 as a standard condition: rabbit monoclonal anti-E-cadherin (Cell Signaling Technology, 3195), mouse monoclonal anti-γ-tubulin, (Sigma-Aldrich, 5326), rabbit polyclonal anti-phospho histone H3 (pHH3) (Merck Millipore, 06-570), rat monoclonal anti-pHH3 (Abcam, ab10543), rabbit polyclonal anti-phospho-myosin light chain (pMRLC) (Abcam, ab2480), rabbit polyclonal anti-Pax2 (Thermo Fisher Scientific, 71-6000), rabbit polyclonal anti-Vangl1 (Atlas Antibody, HPA025235), goat polyclonal anti-Vangl2 (Santa Cruz Biotechnology, sc-46561), and mouse monoclonal anti-ZO-1 (Thermo Fisher Scientific, 33-9100). We used paraformaldehyde (PFA) as fixative for anti-E-cadherin, anti-γ-tubulin and anti-pHH3 immunostaining; we used trichloroacetic acid (TCA) as fixative for anti-pMRLC, anti-Vangl1, anti-Vangl2 and anti-ZO-1 immunostaining.

#### Whole-tissue immunofluorescence and fluorescent dye

Dissected epididymides were fixed with 4% PFA in PBS for 2 h at 4°C or for 20 min at 37°C, or with 2% TCA in Ca^2+^- and Mg^2+^-free PBS for 20 min at 4°C, depending on target epitopes. The samples were then blocked by incubation in 10% normal goat serum (NGS) (Abcam, ab156046) or 10% normal donkey serum (Abcam, ab166643) diluted in 0.1% Triton X-100/PBS, depending on the secondary antibody species, for 3 h at 37°C. The samples were treated with primary antibodies overnight at 4°C, and subsequently incubated with secondary antibodies conjugated to either Alexa Fluor 546 or Alexa Fluor 647 (1:1000, Thermo Fisher Scientific) overnight at 4°C. We used TRITC-conjugated phalloidin (0.3 μg/ml, Merck Millipore, FAK100) and Hoechst 33342 (5 μg/ml, Thermo Fisher Scientific, H3570) for the visualization of F-actin and DNA, respectively.

#### Volumetric fluorescence imaging

We mounted samples with 10 μl of 1% agarose gel on a glass-based dish (Greiner Bio-One, 627871) for stable imaging. Then, the samples were immersed with the BABB solution (benzyl-alcohol and benzyl-benzoate, 1:2) or CUBIC2 solution for optical clearing (Hirashima and Adachi, 2015; Susaki et al., 2014; Yokomizo et al., 2012). Finally, we obtained 8-bit volumetric fluorescence images using the confocal laser-scanning platform Leica TCS SP8 equipped with the hybrid detector Leica HyD. We used objective lens magnifications of ×20 [numerical aperture (NA)=0.75, working distance (WD)=680 μm, HC PL APO CS2, Leica], ×40 (NA=1.3, WD=240 μm, HC PL APO CS2, Leica) or ×60 (NA=1.4, WD=140 μm, HC PL APO CS2, Leica).

#### Explant organ culture

The epididymides were surgically excised at a region of the vas deferens close to the tail of epididymis, and those with testis were dissected from the embryonic body. Samples were then placed on a hydrophilic polytetrafluoroethylene organ culture insert with a pore size of 0.4 μm (Merck Millipore, PICM01250), which was preset in a 35 mm Petri dish (Thermo Fisher Scientific, 153066) filled with a culture medium. The culture medium we used was Dulbecco's Modified Eagle's medium (Nacalai Tesque, 08489-45), containing 10% fetal bovine serum (Thermo Fisher Scientific, 12483) and 1% penicillin-streptomycin mixed solution (Nacalai Tesque, 26253-84) at 37°C under 5% CO_2_. The samples were cultured in an air-liquid interface; the total amount of culture medium was 800 μl for the Petri dish.

#### Small-molecule inhibitors

The following inhibitors were used: blebbistatin (Merck Millipore, 203391), Y-27632 (Merck Millipore, SCM075) and mitomycin C (Nacalai Tesque, 20898-21).

#### Live imaging

The organ culture conditions were as described above with the following two minor modifications. First, we used a 35 mm glass-based dish (Iwaki, 3910-035) with 700 μl of the culture medium in the dish. Second, we adhered each supporting leg of the organ culture insert on the dish with 20 μl of 1% agarose gel. This preparation prevents the culture insert from sliding during the live imaging. We used an incubator-integrated multiphoton fluorescence microscope (Olympus) using a ×25 water-immersion lens (NA=1.05, WD=2 mm, Olympus). Imaging conditions were as follows: excitation wavelength: 945 nm (Mai-Tai DeepSee eHP, Spectra-Physics); AOTF: 3.5%-1.5%; scan size: 1024×320 pixels; scan speed: 10 μs/pixel; *z*-axis interval: 1 μm; time interval: 5 min; magnification: ×2.

#### Tissue compression assay

For the tissue compression assay, we used a manual stretch/compression device (STREX, STB-10) with a polydimethylsiloxane (PDMS) chamber (STREX, STB-CH-04), and carried out the following steps. First, we exposed the PDMS chamber to plasma arc for 1 min using a desktop vacuum plasma processing device (STREX, PC-40) for hydrophilic treatment to enhance the adhesion between the chamber and collagen gel into which the epididymides were embedded. Next, we immediately placed isolated epididymides onto the hydrophilized PDMS chamber in its 50% stretched state, and filled the chamber with 500 μl of type I collagen (Nitta Gelatin, Cellmatrix Type I-A), followed by gelation for 10 min at 37°C. Then, we added 1 ml of the culture medium into the chamber, and the epididymides with the collagen gel were compressed by relaxing from the 50% stretched state around 0.2 mm/s. No relaxing of the stretched chamber was regarded as control treatment. Finally, the samples were incubated for 10 min for the morphological confirmation analysis and 20 min for the western blotting analysis at 37°C under 5% CO_2_. In the lateral compression assay, the epididymal tubules were deformed along the circumferential axis, and the circumferential length of the individual cells became smaller. We believe this is due to the weight of the culture medium filling the upper layer of the gel, as this does not happen without the culture media.

For the immunoblotting assay, we extracted total protein from each treatment, and adjusted the amount such that it was the same across treatments for SDS-PAGE as described in the next section. We confirmed that there were no significant differences in GAPDH expression and in MRLC expression across treatments. The activities were each normalized to the mean values of the control group.

#### Total protein extraction and western blotting

For total protein extraction, dissected epididymides were immersed in SDS-free RIPA buffer with protease inhibitor cocktail (Nacalai Tesque, 08714) supplemented with the EDTA-free phosphatase inhibitor cocktail (1:100, Nacalai Tesque, 07575), and were disrupted by an ultrasonic cell disruptor (Microson). The lysates were placed onto ice for 20 min and were centrifuged at 13,000 ***g*** for 10 min at 4°C. The protein concentration of the supernatant was determined by bicinchoninic acid assay.

The lysates were prepared for SDS-PAGE by adding 2× Laemmli sample buffer (Bio-Rad, 161-0737) with 2-mercaptoethanol (Bio-Rad, 161-0710) and by boiling at 96°C for 5 min. Next, the lysates containing approximately 5 μg of proteins were loaded into each lane of Mini-PROTEAN precast gels (Bio-Rad, 4569035), and electrophoresis was carried out in Tris/glycine/SDS running buffer (Bio-Rad, 1610732) at constant 150 V for 35 min. Then, the proteins were blotted onto 0.2 μm polyvinylidene difluoride membrane (Bio-Rad, 1704272) in HIGH MW mode (1.3 A, 25 V for 10 min) of the Trans-Blot Turbo Transfer System (Bio-Rad, 170-4155) for ROCK1 detection and in the LOW MW mode (1.3 A, 25 V for 5 min) for others.

The blotted membranes were then immersed in 15% H_2_O_2_/Tris-buffered saline (TBS) solution for 30 min at room temperature for blocking endogenous peroxidase followed by blocking with 5% NGS at 37°C for 60 min. For immunoblotting, the membranes were incubated with primary antibodies diluted in 0.1% TBS/Tween-20 at 4°C overnight. The concentrations of antibodies used were 1:100,000 for mouse monoclonal anti-GAPDH (Wako, 015-25473), 1:500 for rabbit polyclonal anti-myosin light chain 2 (Cell Signaling Technology, 3672) and mouse monoclonal anti-phospho-myosin light chain 2 (Cell Signaling Technology, 3675), and 1:2000 for rabbit monoclonal anti-ROCK1 (Abcam, ab45171). Then, the membranes were incubated with diluted secondary antibody solutions in TBS/Tween-20 at 37°C for 60 min; the concentration was 1:50,000 for goat anti-rabbit IgG conjugated to horseradish peroxidase (HRP) (Santa Cruz Biotechnology, sc-2004) and goat anti-mouse IgG conjugated to HRP (Santa Cruz Biotechnology, sc-2005). Finally, protein bands were detected using the Amersham ECL Select Western Blotting Detection Reagent (GE Healthcare, RPN2235), and were scanned with ImageQuant LAS 4000 (GE Healthcare).

### Quantification

#### Cell division orientation

We first performed co-immunolabelling for γ-tubulin and pHH3 of six different epididymides to detect MTOCs of mitotic cells as described above with a minor modification. That is, the samples were incubated in 10 mM sodium citrate buffer including 0.1% Triton X-100/PBS for 20 min at 80°C for heat-induced retrieval of γ-tubulin epitope before blocking treatment. Next, we obtained volumetric images of 1024×256 pixels as described above using ×40 lens with *z*-axis interval of 1 μm. Third, we manually measured three angles, α_1_, α_2_ and α_3_, for each mitotic cell to define a local coordinate system, O′, the origin of which corresponds to the centre position of the mitotic cell. Each orthogonal basis of the O′ coordinate was defined as follows: longitudinal direction of epididymal tubule (*x*′), surface normal of epididymal tubule (*z*′), and orthogonal direction for both *x*′ and *z*′ according to the right-handed system (*y*′). Then, we manually measured the positions of the two γ-tubulin-positive dots (MTOCs) in a pHH3-positive cell, and obtained two angles, φ and θ, in a local sphere coordinate resulting from coordinate transformation into the O′ system. We measured all pHH3-positive cells we could recognize. Finally, we selected samples under a criterion that the distance between the two γ-tubulin-positive dots was more than 1.5 μm. We regarded the samples for which φ was less than 40° as cells dividing in parallel to the surface of epididymal tubule.

#### Apical junction morphology and pMRLC/VANGL signal intensity

We performed co-immunostaining for ZO-1 and either pMRLC, F-actin or VANGL of eight different epididymides as described above with a minor modification: the dilution ratio of anti-pMRLC was 1:400 to prevent saturation binding. We obtained volumetric images of 1024×256 as described above using ×60 lens with *z*-axis interval of 0.3 μm. Then, we separated the images into smaller ones, around 200×200 pixels (≈36 μm), so that the slope of epididymal tubule could be regarded as a linear line. The digital image processing was performed as follows. First, images for ZO-1 were filtered slice by slice with a 2D median filter (3×3 pixels) followed by a 3D Gaussian filter with a standard deviation of 3 pixels to reduce undesired noise. Next, the filtered images were binarized using the 3D maximum entropy thresholding method (Kapur et al., 1985), and the maximum binarized component of 26-connected pixels was regarded as an apical junction network. Then, we performed 3D skeletonization process for the images of apical junctions, and obtained a graph network including nodes and edges of skeletonized voxels utilizing distributed MATLAB codes with modifications (Kerschnitzki et al., 2013). Finally, to extract the skeleton of apical cellular junctions, we screened out the skeletonized edges to avoid over-skeletonization results in the following three criteria: (1) the edge length between two nodes is less than 10 μm, (2) the edge length is shorter than twice the linear length between the two nodes linked by the corresponding edge, (3) the number of edges from a node is more than three. The screening process under these criteria resulted in 96% remaining from the total number of raw skeletonized edges. The relative angle of apical junction edges against the longitudinal axis of epididymal tubule was calculated as ϑ_*j*_ = |cos^−1^(**ĝ**_*j*_· **l̂**)| where *j* is an index of apical edges, **ĝ** is a unit vector of apical edges, and **l̂** is that of the longitudinal axis of tubule. For signal quantification, we extracted the target signal intensity on the apical cellular junctions by using a thickened skeleton of apical junctions with a dilation operator (3×3×3 voxels), and calculated average values for each edge of thickened apical junction skeletons.

#### Tubule diameter in the inhibitor assay

First, we performed immunostaining for E-cadherin for each inhibitor treatment to visualize the epididymal tubule as described above, and obtained the volumetric images of 512×512 pixels using a ×20 lens with *z*-axis interval of 5 μm. We utilized the built-in module of a software platform, Leica Suite X, to create a montage image of whole epididymis. Then, we manually extracted epididymal tubules from maximum intensity projection images by erasing the efferent ductules and noise pixels caused by non-specific staining. From the preprocessed images, we obtained the centre line of the epididymal tubule by applying the built-in skeletonization algorithm in MATLAB for the binary images obtained with arbitrarily determined thresholds, and finally calculated the diameter along the centre line of tubule.

#### Cell intercalation rate for tube elongation (CITE)

We first obtained time-lapsed volumetric images for epithelial dynamics from the head region of four different samples at E15.5 by live imaging as described above, and collected time series data for a group of cells around mitotic cells. In particular, we sampled as many cells as possible on an image plane perpendicular to the optical axis from the image data, and focused on cross-sections between the apical and basal part of cells. We defined cytokinesis as the origin of time for our analysis, and analysed data on cell intercalation dynamics with a time interval of 10 min (Δ*t*=10 min). It was hard to find a cluster of cells unaffected by cell division within our observation windows because the cell division occurs incessantly.

Next, based on an earlier study, we calculated the cell intercalation strain rate tensor **L**_**I**_^*t*^ at time *t* as follows:
(1)
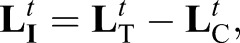
where **L**_T_^*t*^ is a local tissue velocity gradient tensor, and **L**_C_^*t*^ is a cell shape strain rate tensor (Blanchard et al., 2009). Because our analysis was performed in planar, the tensors are 2×2 matrices. For calculation of the local tissue velocity gradient tensor **L**_T_^*t*^, we specified one or two neighbouring cells of a daughter cell from a mitotic cell that was located on an extension of the division axis as central cells, and marked the adjacent cells of the central cells, including the central cells and the daughter cell, as domain cells. Then, we manually traced each one of *n* domain cells to obtain cell areas, cell shapes with the best fit ellipses, and cell centroids **r**_*j*_^*t*^, where *j* is an index of domain cells (marked cells by yellow lines in Fig. S4). Using cell centroids (**r**_*j*_^*t*^) data and velocity (**u**_*j*_^*t*^) data, the local velocity gradient tensor was obtained by least squares approximations according to the following equation:
(2)
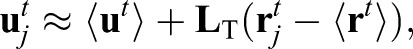

where 〈**r**^*t*^〉=Σ_*j*_ **r**_*j*_^*t*^/*n*, **u**_*j*_^*t*^ = (**r**_*j*_^*t*+Δ*t*^ − **r**_*j*_^*t*^)/Δ*t* and 〈**u**^*t*^〉=(〈**r**^*t*+Δ*t*^〉−〈**r**^*t*^〉)/Δ*t*. 〈·〉 indicates an average within the domain.

For calculation of the cell shape strain rate tensor, **L**_C_^*t*^, we used lengths of principal axes of a fitted ellipse and the angle of the major axis from the longitudinal axis of epididymal tubule to obtain a matrix, **C**_*j*_^*t*^, that represents the cell ellipse. Then, we calculated **L**_C,*j*_^*t*^ from **C**_*j*_^*t*^ and **C**_*j*_^*t*+Δ*t*^, and **L**_C_^*t*^ was calculated by taking the area-weighted average of **L**_C,*j*_^*t*^ within the domain cells. Note that we normalized **L**_C_^*t*^ such that a trace of **L**_C_^*t*^ was equal to that of **L**_T_^*t*^, in the case that the cell intercalation strain rate tensor **L**_T_^*t*^ had zero dilation.

Finally, we obtained the CITE at time *t* (*L*_*ll*_^*t*^) from an element of the cell intercalation strain rate tensor:
(3)
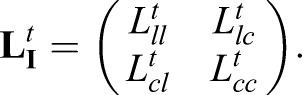
Note that *L*_*ll*_^*t*^ = −*L*_*cc*_^*t*^.

The data were classified into two groups depending on the relative angle of the cell division axis to the longitudinal axis of epididymal tubule – we defined a relative division angle of 0-30° as longitudinal division and that of 60-90° as circumferential division.

### Mathematical modelling

#### Basic formalization of the vertex dynamics model

We employed a vertex dynamics model (VDM), a type of cell-oriented model, to represent multicellular dynamics in epithelial tissues (Farhadifar et al., 2007; Fletcher et al., 2014; Nagai and Honda, 2001; Rauzi et al., 2008). In this model, a cell is geometrically regarded as a polygon/polyhedron, of which vertices are elementary points that constitute the cell shape, and a group of cells can be represented by a set of polygons/polyhedrons shared by neighbouring cells (Fletcher et al., 2013; Honda and Eguchi, 1980; Honda et al., 2004; Nagai and Honda, 2001; Okuda et al., 2013). In this study, we identified apical junctions of the epididymal tubule on which only pMRLC, a marker of tension generation, is localized (Fig. 2A), and modelled the developing single-layered epithelial tube as a flexible multicellular membrane in 3D space according to earlier studies (Du et al., 2014; Osterfield et al., 2013).

In the VDM, the dynamics of position of vertex *i*, **r***_i_*, obey the equation of motion based on the principle of least potential energy *U* as follows:
(4)
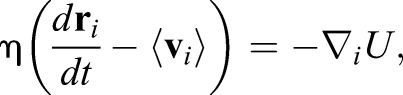
where η is a viscosity coefficient, and 〈**V**_*i*_〉 is the local velocity of vertex *i*, defined as:
(5)
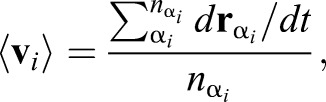
where α_*i*_ is an index for cells contacting to vertex *i*, *n*_α_*i*__ is a number of the cells contacting to vertex *i*, and *r*_α_*i*__ is a centroid of cell α_*i*_ (Mao et al., 2013; Okuda et al., 2015). For a potential energy as a minimum expression to represent epididymal tubule cells, we used the equation:
(6)
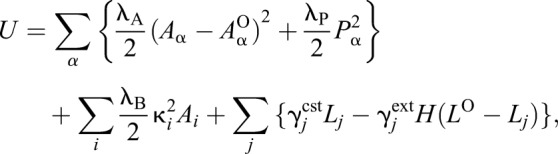
according to earlier studies (Collinet et al., 2015; Farhadifar et al., 2007; Rauzi et al., 2008). α, *i* and *j* each denote an index of cells, of vertices, and of junctions/edges, respectively. The first term represents the cell elasticity; λ_A_ is its coefficient, *A*_α_ is the area of cell α, and *A*_α_^O^ is the target area of cells. The second term represents isotropic actomyosin contractility at the periphery of apical junctions; λ_p_ is its coefficient and *P*_α_ is the perimeter of the cell α. The third term represents bending energy of the flexible epithelial membrane embedded in 3D space; λ_B_ is its coefficient, κ*_i_* is a discrete mean curvature defined at vertex *i* of triangular meshes (Meyer et al., 2003), and *A_i_* is the summation of an area of triangular mesh fractions around vertex *i*, each explained one by one. For calculation of the discrete mean curvature at a vertex, we let the surface of the modelled epithelial membrane be a set of triangular meshes consisting of both the vertex of cells and the centroid of cells. Then, we defined the discrete mean curvature at vertex *i* as:
(7)
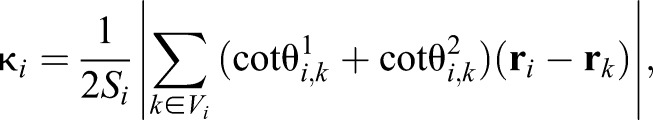
where *k* is an index of the triangular elements included in the set of 1-ring neighbour vertices of the vertex *i*, *V*_*i*_; *S*_*i*_ is the total area of triangular meshes in *V*_*i*_; and θ_*i,k*_^1^ and θ_*i,k*_^2^ are each angle depicted in Fig. S6. See (Meyer et al., 2003) for more details. For calculation of *A*_*i*_, we considered triangles consisting of a vertex and its 1-ring neighbour cell centroids as illustrated in Fig. S6, and then summed all triangles up around the vertex. In earlier studies, bending energy was defined as a summation of the inner product of unit normal vectors to the surfaces between the adjacent cells (Du et al., 2014; Osterfield et al., 2013). This is valid when all mesh sizes are ideally equal (Kantor and Nelson, 1987; Seung and Nelson, 1988); therefore, we introduced the bending energy function based on the discrete curvature. The last term of Eqn 6 represents anisotropic junction constriction/extension; γ_*j*_^cst/ext^ is its coefficient assigned to each edge, *L*_*j*_ is length of edge, *L*^O^ is a target edge length, and *H* is the Heaviside step function. γ_*j*_^cst/ext^ = 0 for the simulation shown in Fig. 7A. The details of this term are described later.

#### Normalized form of the model

By introducing a characteristic cell size, *A*_0_, the equations were transformed into a nondimensionalized form to reduce the number of parameters as:
(8)
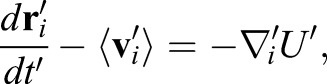
where *t*′=*t*λ_A_*A*_0_/η, 
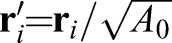
, 
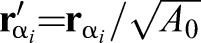
, 
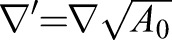
, *U*′=*U*/(λ_A_*A*_0_^2^); and:
(9)
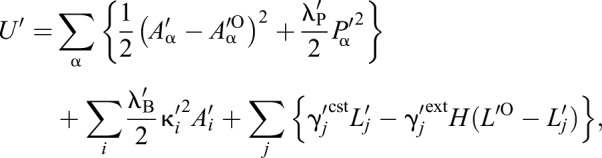


where *A*′_α_=*A*_α_/*A*_0_, *A*_α_^′O^=*A*_α_^O^/*A*_0_, 
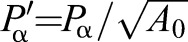
, 
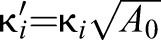
, *A*′_*i*_=*A_i_*/*A*_0_, 
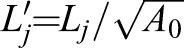
, 
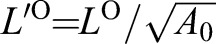
, λ′_P_=λ_P_/(λ_A_*A*_0_), λ′_B_=λ_B_/(λ_A_*A*_0_^2^), γ_j_^′cst/ext^=γ_j_^cst/ext^/(λ_A_*A*_0_^−3/2^). In addition, we introduced a normalized simulation time defined as τ′=*t*′/*T* ^cycle^, where *T* ^cycle^ is an average cell cycle length, which will be explained. We used these variables and Eqns 8 and 9 for the numerical simulations in this study.

#### Cell intercalation, proliferation, and simulation conditions

For the cell intercalation, we assumed that edge rearrangement occurred when the normalized length of the edge became smaller than 0.05 and the edge length was set to be 0.1 after the edge rearrangement. See Fletcher et al. (2013) for details of the edge rearrangement.

For the cell proliferation, we introduced the average cell cycle length *T* ^cycle^, and set the cell cycle length of each cell, *T*_α_^cycle^, to obey the normal distribution of which the mean is *T* ^cycle^ and the standard deviation is 5% of the mean, i.e. *T*_α_^cycle^ ∼ *N*(*T*^cycle^, 0.05*T*^cycle^). Also, we introduced the normalized cell cycle time of each cell as τ_α_′^cycle^ = *t*_α_′^cycle^/*T*_α_^cycle^, where *t*_α_′^cycle^ is the nondimensionalized cell cycle time of cell α. τ_α_′^cycle^ at *t*′=0 was assigned within 0 to 0.95 in a uniform distribution. As the average cell cycle length in the epididymal tubule can be roughly estimated as 24 h and cell volume tends to increase within the apical surface of the epididymal tubule from 50 to 100 min before the cytokinesis, we simply modelled the cell dynamics during the cell cycle as *A*_α_′^target^ = 1 for 0 ≤ τ_α_′^cycle^ < 0.95, *A*_α_′^target^ = 20τ_α_′^cycle^ − 18 for 0.95 ≤ τ_α_′^cycle^ < 1, and the cell divides to return the *A*_α_′^target^ of daughter cells to 1.The type of cell division orientation was set as follows: circumferential/longitudinal division with a uniform distribution in the range of ±0.05π from the circumferential/longitudinal axis of tube, and non-biased division with a uniform distribution in the range of θ as defined by the local coordinates of the cell.

As the initial condition for the simulation, we designed a virtual tube composed of 14 cells along the circumferential direction based on our observation and of 30 cells along the longitudinal axis for extensive numerical simulations. The cross-sectional centre of the tube was set at *y*=0 and *z*=0 (global coordinates) and the tube was put along the *x* axis. For the boundary condition, the following energy was added to the original potential energy, *U*′, at the tip cells of the tube:
(10)
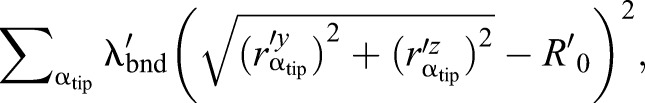
where α_tip_ is an index for the tip cells of the tube; λ′_bnd_ is its coefficient; *R*′_bnd_ is the normalized mean radius of the tube to a characteristic cell length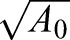 in the initial setting; and *r*_αtip_^′*y*^ and *r*_αtip_^′*z*^ each represent the position of the centre of cells α_tip_ on the *y-* and *z*-axis relative to 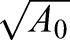. This energy restricts displacement of the cells α_tip_ in the radial direction of the tube, which imitates physical constraints by the vas deferens and the efferent ducts as the boundary tissues of the epididymal tubule. We set λ′_bnd_=0.1 in the simulations. The results did not vary qualitatively even without the boundary energy term Eqn 10.

#### Mechano-responsive and scheduled regimes

Here, we explain the last two terms of Eqn 9: the anisotropic edge constriction and extension after the cell intercalation. We introduce a state variable assigned to each cell, Θ_α_={0,1,2}; Θ_α_=0,1,2 represent a naïve state (Fig. 7D, white), edge-constriction state (Fig. 7D, red) and edge-extension state (Fig. 7D, blue), respectively. For all cells, Θ_α_=0, γ_*j*_^′cst^=0 and γ_*j*_^′ext^=0, unless otherwise noted. In our simulation, the following procedures were conducted for both the ‘mechano-responsive’ and ‘scheduled’ regimes. First, cells (α′) that might become a constricting cell were selected and we let the state variable of the cell Θ_α′_=1; the way of selecting cells depends on each regime, i.e. the mechano-responsive or the scheduled regime. Second, one of the edges orientated with the circumferential axis of the tube (the angle θ*_j_* is within 70°–90°) was selected among all edges of the cells α′, and the edge was indexed as *j* ′. Then, the state of cells including the edge *j* ′ was set as Θ_α′_=1. Third, constriction of edge *j* ′ was performed according to a schedule as shown in Fig. S7A; we set the edge to keep constricting in a period τ′^cst^ via transition period τ′^trns^ from the starting point of edge constriction based on an assumption of the phosphorylation process of pMRLC. The maximum value of γ_*j′*_′^cst^ was set to be γ′^cst^. Θ_α′_ and γ_*j*′_′^cst^ return to 0 at the end of the edge constriction process. In this study, the parameter dependence of τ′^cst^ and that of γ′^cst^ were examined and determined, and we set τ′^trns^=0.02. Finally, if the edge arrangement occurred during the process of edge constriction (Θ_α′_=1), then the state of cells having the rearranged edge changes to Θ_α′_=2 and the edge becomes an extension mode, in which the rearranged edge converges to a constant target length; γ_*j*′_′^cst^=0, γ_*j*′_′^ext^=0.01 and *L*′^O^=0.1. We set the period of edge extension as τ′^ext^=0.02. Θ_α′_ and γ_*j*′_′^ext^ return to 0 after the process of edge extension. Our implementation for the edge extension process is based on an earlier report that the junction after the cell intercalation grows due to local polarized force driven by medial actomyosin contraction (Collinet et al., 2015).

In order to select the cells α′ in the first step described above, we adopted two regimes: mechano-responsive and scheduled. For the mechano-responsive regime, we supposed that the epididymal tubule cells could receive mechanical stress provided by adjacent cells, and a stress tensor in cells α in the local cell coordinate system *O′* was defined as:
(11)
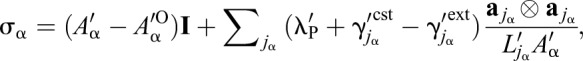
where **I** is a unit tensor, *j*_α_ is an index of the edges composing the cell α, **a** is a vector representing the relative position of two vertices composing the edge *j*_α_ and ⊗ indicates the tensor product (Batchelor, 1970; Ishihara and Sugimura, 2012; Sugimura and Ishihara, 2013). Denoting a diagonal element of the tensor σ_α_ corresponding to the longitudinal axis of the tube and that to the circumferential axis as σ_α_^long^ and σ_α_^circ^, we then defined the cell tension anisotropy as:
(12)
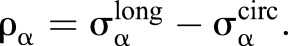
Note that the cell tension anisotropy ρ_α_ is a time-dependent variable. In the mechano-responsive regime, the cells were assigned as α′ when ρ_α_was over a threshold ρ*, of which parameter dependency for the resulting tube morphology was examined (Fig. S7).

For the scheduled regime, the cells α′ were randomly selected but timing of the selection was predetermined according to the schedule; Fig. 7F shows the fraction of cells having constricting edges to all tube cells in the mechano-responsive regime (*n*=10, grey), and its mean (black). In our simulation, we used the following logistic function (blue) by fitting the mean curve with the least-squared method:
(13)
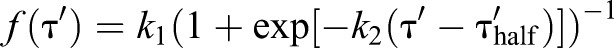
with *k*_1_=0.1, *k*_2_=0.01 and *k*_3_=0.17**.**

#### Morphological and mechanical quantities in simulation

We used the following morphological quantities in the simulation: (1) tube length, i.e. the longitudinal linear length between distal edges of the tube, (2) tube diameter, i.e. the mean diameter of the circumferentially averaged diameter through the centre line of the tube, and (3) curvedness, i.e. the averaged curvature of the vertices throughout the tube defined as Σ_*i*_*k*′_*i*_*A*′_*i*_/Σ_*i*_*A*′_*i*_.

For the cell tension anisotropy ρ_α_, refer to Eqn 12. For the tube tension anisotropy, the stress tensor in the tube was defined as:
(14)
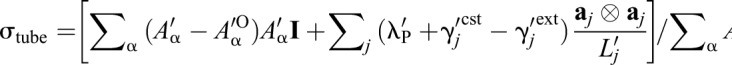
according to earlier studies (Batchelor, 1970; Ishihara and Sugimura, 2012), and using its diagonal element σ_tube_^long^ and σ_tube_^circ^, we introduced:
(15)
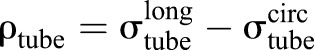
as the tube tension anisotropy. σ_tube_^long^ and σ_tube_^circ^ each correspond to the tube longitudinal tension and the tube circumferential tension.

#### Determination of parameter values

The parameter values used in this study were: (1) λ′_P_=10^−2^, λ′_B_=10^0.75^, (2) *T*^cycle^ = 10^3.75^, (3) ρ*=0.1, γ′^cst^=1, and τ′^cst^=0.167, unless otherwise noted. For (1), we determined the values for which there was less variations at *t*′ = 1000 from the initial configuration. Note that no proliferation was implemented for this analysis. For (2), we examined the parameter dependence of the cell proliferation rate 1/*T* ^cycle^ to the morphological and the mechanical quantities at τ′=1. The results show that the cell division orientation affected the tube shape (length and diameter) to a lesser extent at a faster cell proliferation rate. In addition, the smooth surface of the tube was not maintained if the cell proliferation rate was greater than 10^−3.5^. This is because the cell proliferation rate is faster than the relaxation time of the tube in the dynamics. We determined that *T*^cycle^ = 10^3.75^. For (3), we examined the parameter dependence of ρ*, γ′^cst^ and τ′^cst^ to the tube shape and its variance at τ′=1. We first determined ρ*=0.1 because the coefficient of variation (CV) became larger at ρ*=0.08 and there was less difference in the mean values of the variation of γ′^cst^ at ρ*=0.125. Then, we determined γ′^cst^=1 because this point is at the boundary of the mean and the CV. Finally, as for τ′^cst^, we set the value arbitrarily because there was less difference between the values.

### Analysis

#### Statistical hypothesis testing

Exact information of the statistical tests, sample sizes, test statistics, and *P*-values were described in the main text. Statistical hypothesis testing was performed according to Zar (2014). *P*-values of less than 0.05 were considered to be statistically significant in two-tailed tests, and were classified as four categories: *P<0.05, **P<0.01, ***P<0.001, and n.s. (not significant, i.e. *P*≥0.05).

#### Replicates

All replicates in this study are biological replicates. Experiments were reproduced more than three times.

#### Graphs

Except for boxplots, graphs were plotted as mean±s.d. For boxplots, the central horizontal bar indicates the median, and the bottom and top edges indicate the 25th and 75th percentiles, respectively. The vertical bars extend to the most extreme data points not considered outliers, and the outliers are omitted. The boxplots were drawn using MATLAB (MathWorks).

#### Software

For digital image processing, we used MATLAB (MathWorks) and ImageJ (National Institute of Health). For graphics, we used MATLAB (MathWorks), R (GNU project), Gnuplot (Free Software), and Paraview (Kitware). For statistical analysis, we used MATLAB (MathWorks) and R (GNU project).

## Supplementary Material

Supplementary information
