# Supplementary material for: Polarized cellular mechano-response system for maintaining radial size in developing epithelial tubes
Source: Development. 2019 Dec 2;146(23):dev181206. doi: 10.1242/dev.181206 (PMC6918744; doi:10.1242/dev.181206)
Supplement: Supplementary information [file develop-146-181206-s1.pdf]

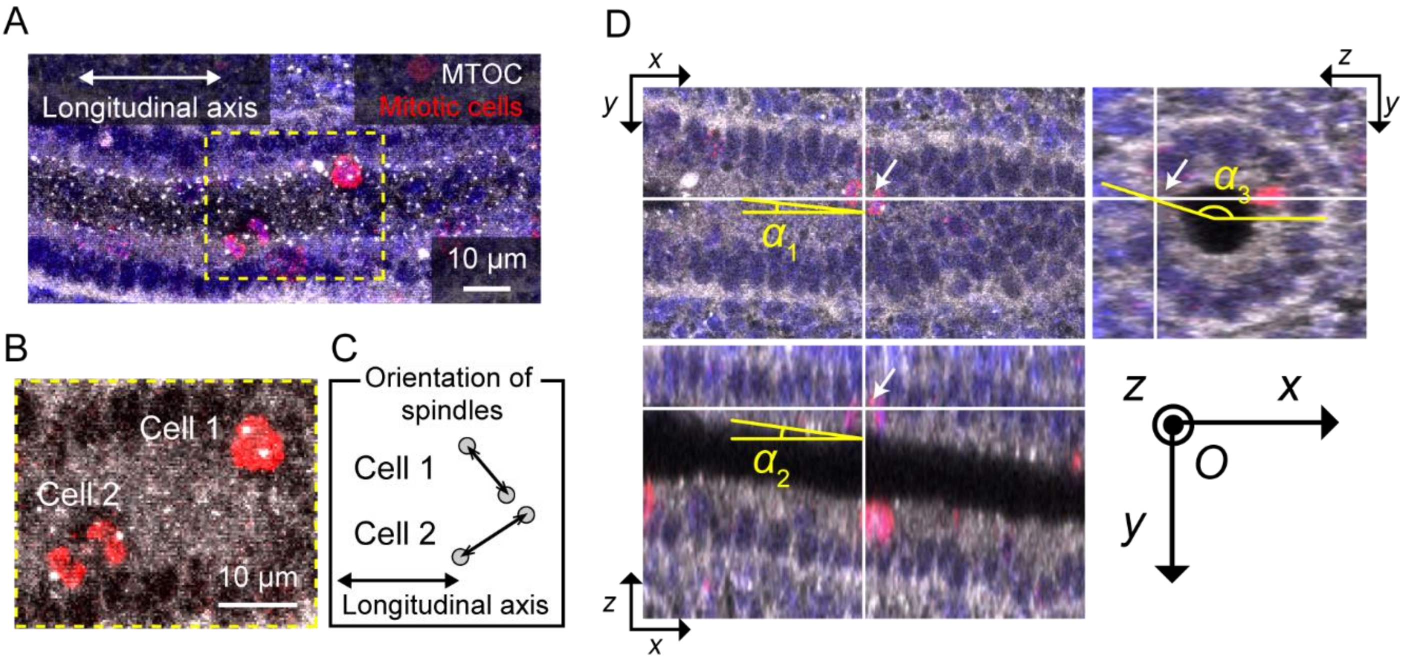

Figure S1. Quantification of spindle or cell division orientation in tubes, corresponding to Figure 1

(A) Maximum intensity projection (MIP) of immunostained images for the pHH3 (mitotic cells, red) and γ-tubulin (MTOCs, white). Scale bar, 10 μm. (B) Magnified view of the dotted square in (A), displaying the two cells divide in different orientations. The image is the same as Fig. 1B. Scale bars, 10 μm. (C) Schematics of (B) for spindle orientation. (D) Measurement of spindle orientation in the tubule. Arrows indicate the center position of the mitotic cell. Using the three angles a_1, a_2, and a_3, the observation coordinate O is transformed into the local coordinate O' in the Fig. 1B.

Development • Supplementary information

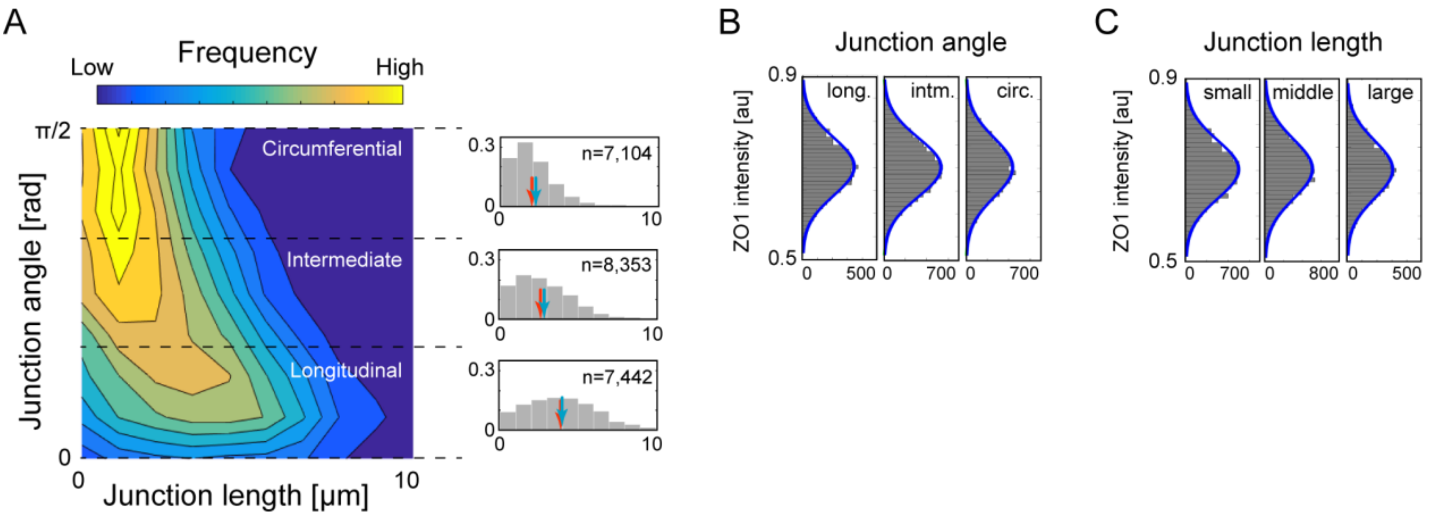

Figure S2. Quantitative analysis of the cell-cell junction, corresponding to Figure 2
(A) Frequency map on junction length ℓ and angle θ (left). The data is summarized as histograms in the angle category (Circumferential, Intermediate, and Longitudinal), respectively (right). The red/blue arrow indicates median/mean. The circumferential junction length is shorter than the longitudinal junction length. n=22,899. (B and C) Relation between the ZO1 intensity and the junction angle/length, representing a control for active myosin profiles.

Development • Supplementary information

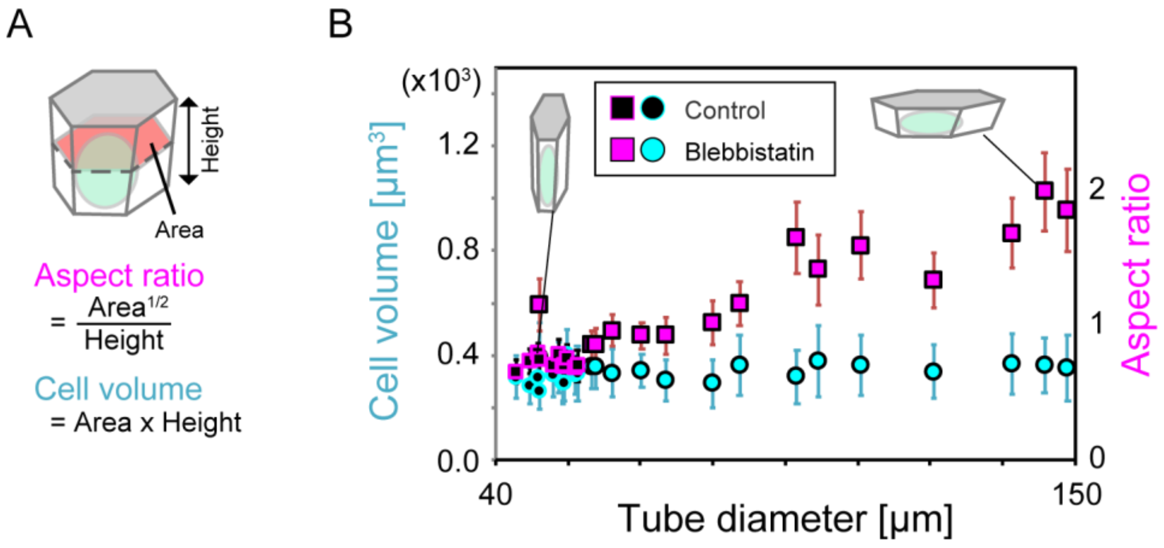

Figure S3. Cell shape and volume in inhibiting actomyosin constriction, corresponding to Figure 3

(A) Schematics for two quantities, aspect ratio and cell volume. The aspect ratio is defined as squared cell area divided by the cell height. (B) Cell volume and aspect ratio in various tube diameter in the Blebbistatin treatment. Although the aspect ratio of cell shape varies in the treatment, the cell volume does not change. *n*=20 from 3 different embryos.

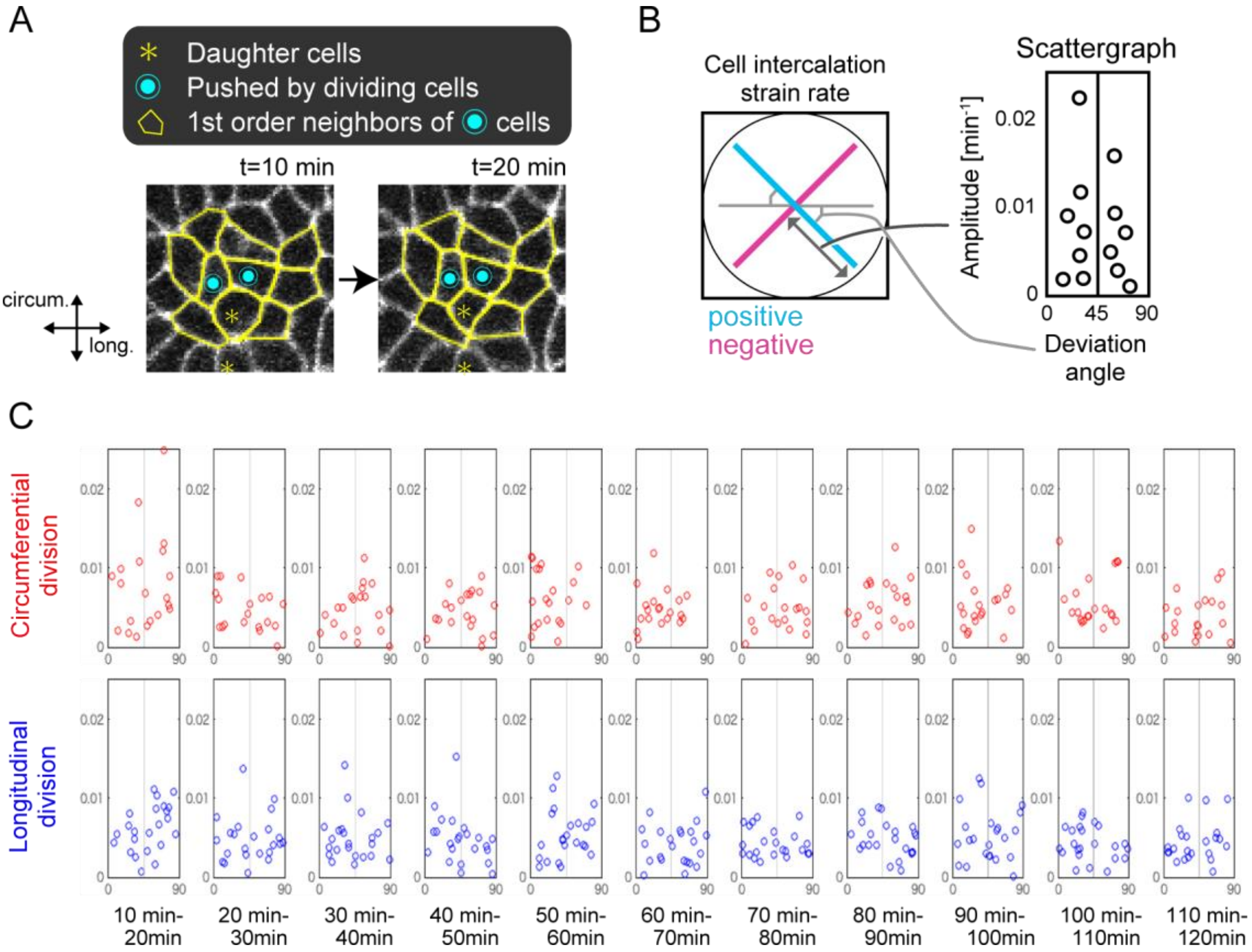

Figure S4. Measurement of cell intercalation strain rate from the live imaging data, corresponding to Figure 4

(A) Illustration for target cells to calculate the cell intercalation strain rate. See the Materials and Methods (II-iv) for the details. (B) A representative example for graph of the cell intercalation strain rate. Orthogonal lines in the left represent principal strain rate. The amplitude and angle deviation of the positive principal strain rate (blue) in the left are expressed in the right angle-amplitude graph. (C) Time course of the cell intercalation strain rate as in the form of angle-amplitude graph for each type of cell division orientation: circumferential cell division (n=20) and longitudinal one (n=23).

Development • Supplementary information

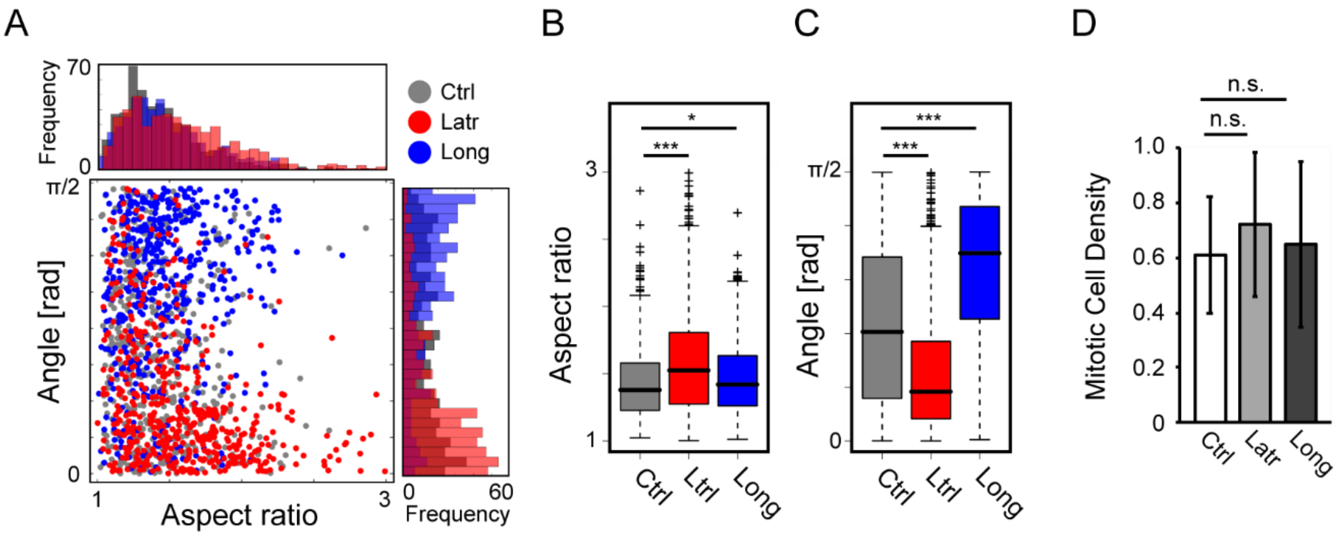

Figure S5. Impact of cell configuration by compression assay
(A) Scatter plot and histograms for the aspect ratio and the major axis angle for each treatment: control (gray), lateral compression (red), and longitudinal compression (blue). *n*=500 from 5 embryos. (B) The aspect ratio in the treatments. (C) The major axis angle in the treatments. The uniaxial compression deforms the cell along the direction (Kruskal-Wallis test, $P<0.05$). (D) Mitotic cell density of the epididymal tubes in the mechanical compression. The compression did not influence the number of cell mitosis (Kruskal-Wallis test, $P \geq 0.05$). *n*=6.

Development • Supplementary information

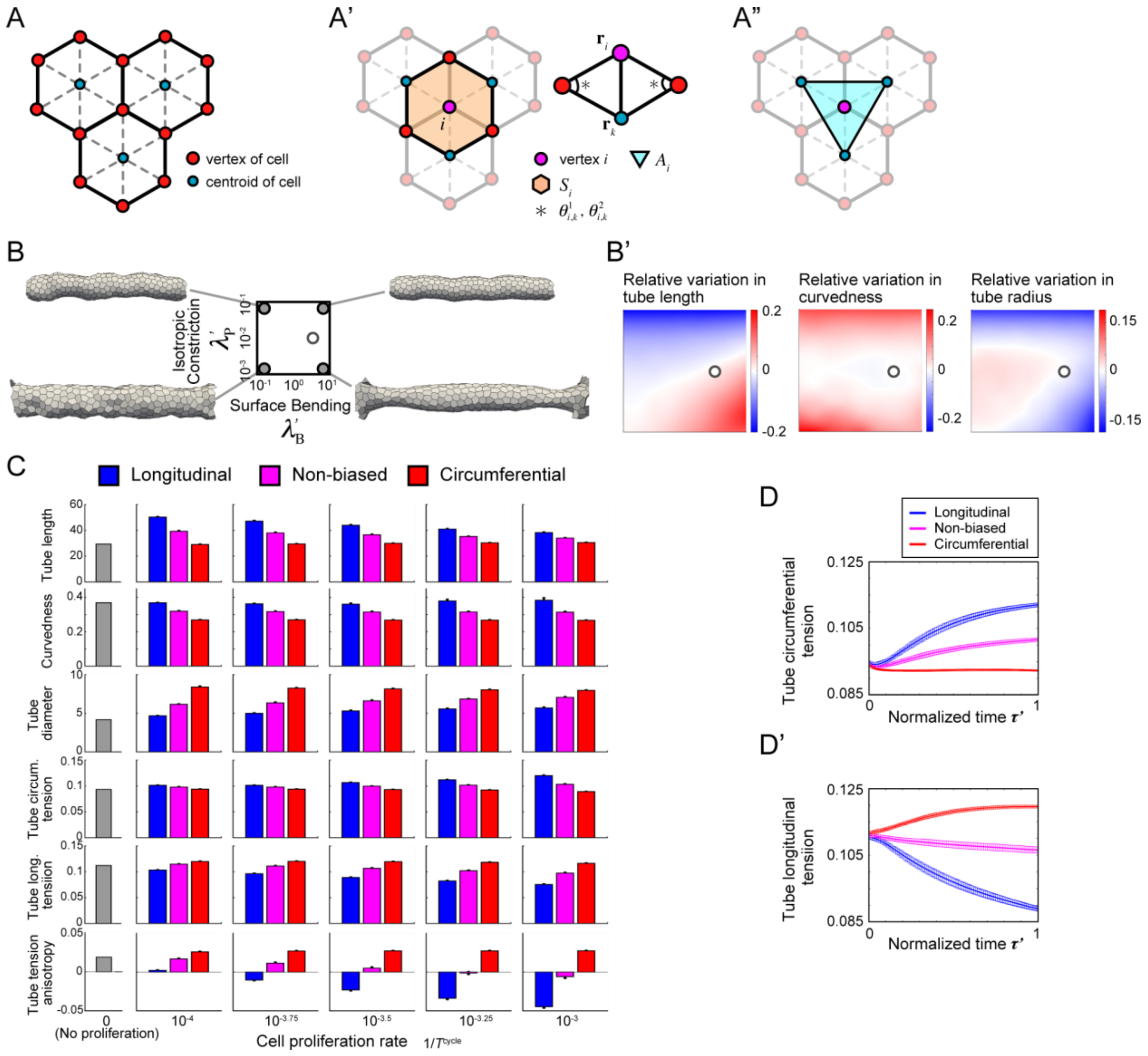

Figure S6 Preparation for the simulation without anisotropic junction constriction, corresponding to Figure 7

(A-A”) Schematics for the explanation of discrete curvature in the vertex model. See the Materials and Methods (III-i) for the notations. (B and B’) Diagram of generated tube morphology for parameters, the isotropic constriction and the surface bending, and relative variation of morphological quantities in the absence of cell proliferation. Tubes are visualized in the four corners of parameter space (filled circles). Open circles represent parameter values used in this study. The axes labels are shown in B. (C) Parameter dependence of morphological and mechanical quantities for cell division orientation and cell proliferation rate. Color represents the type of cell division orientation. (D and D’) Time course of tube circumferential tension and that of tube longitudinal tension. See the Materials and Methods (III-vi) for the definition of tube circumferential/longitudinal tension.

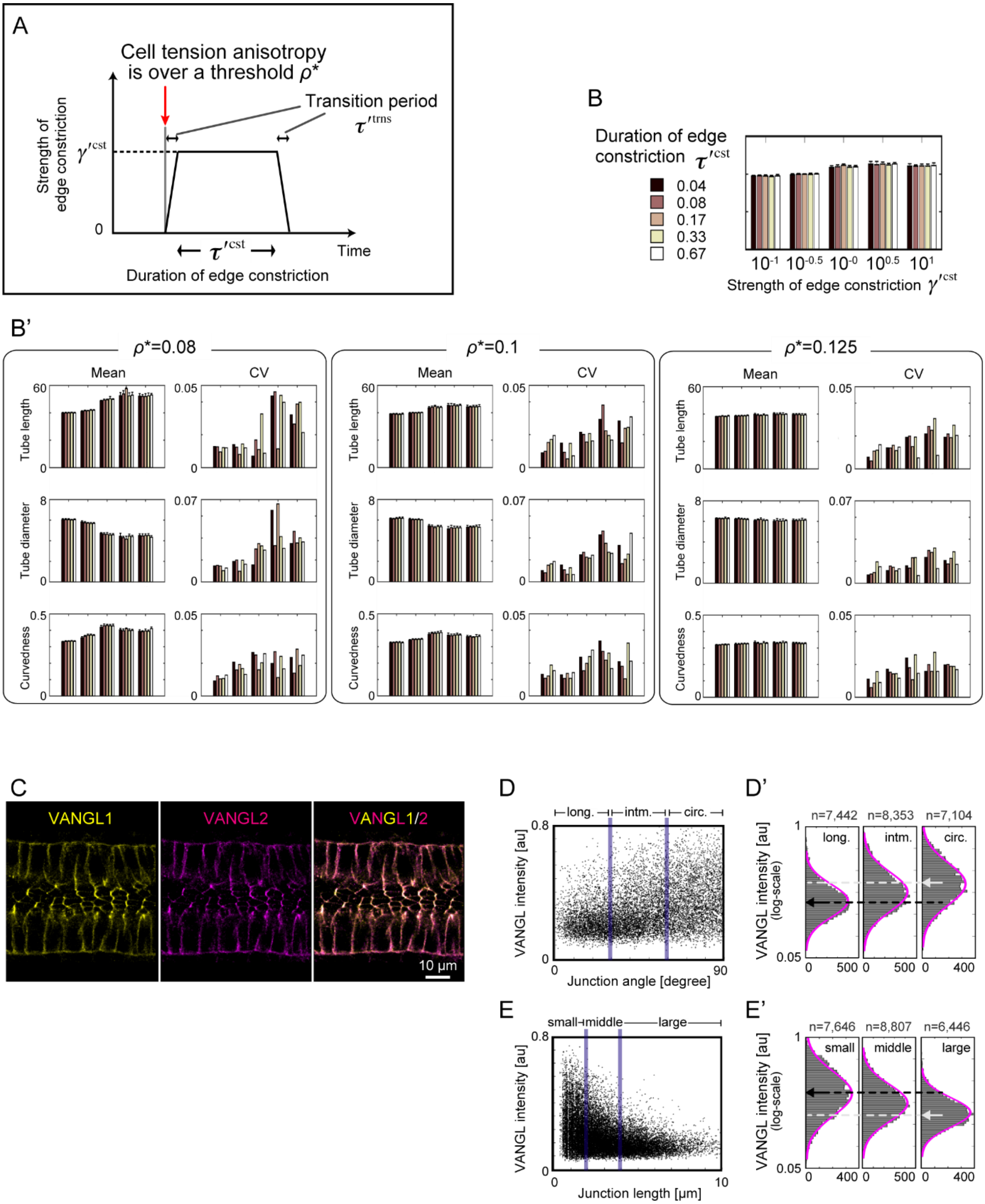

Development • Supplementary information

Figure S7. Models and a PCP component distribution for the mechano-responsive regime, corresponding to Figure 7

(A) Schematics for the model of mechanoresponse. See the Materials and Methods (III-iv) for the details. (B and B') Parameter dependence of tube morphology. The referenced graph is shown in the upper right (B). CV: coefficient of variance. See the Materials and Methods (III-vi) for determining the parameter values. *n*=10. (C) Spatial distribution of polarity proteins Van like protein (VANGL) 1 and 2. Immunofluorescence for VANGL1 and VANGL2 demonstrates that their co-localizations are concentrated on circumferential apical junctions of epididymal tubule. (D-E') Relation between the VANGL1 intensity and the junction angle/length. The samples were categorized into 3 groups, and summarized as histograms on the logarithmic scale (D' and E'). Black arrows represent the mean intensity in the longitudinal (long.)/small group, and gray arrows represent that in the circumferential (circ.)/large group. *n*>7000 from 8 embryos.

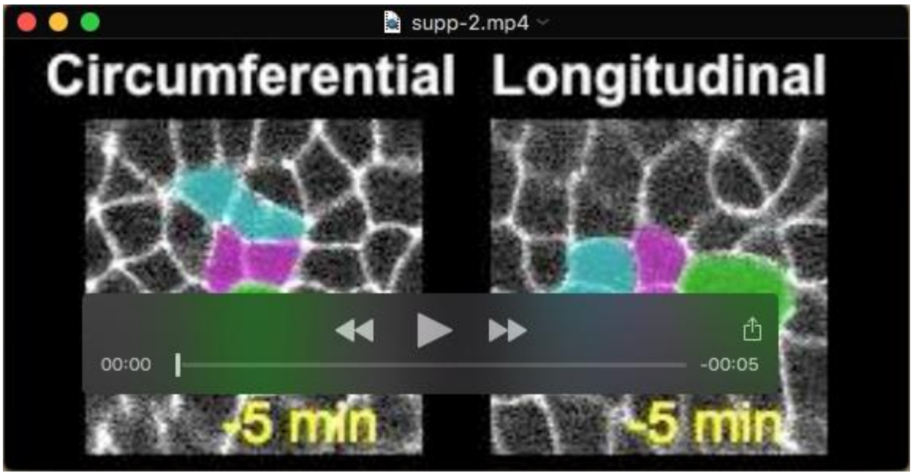

Movie 1. Live imaging for multicellular behaviors in developing epididymal tubes, corresponding to the Figure 4

Development • Supplementary information

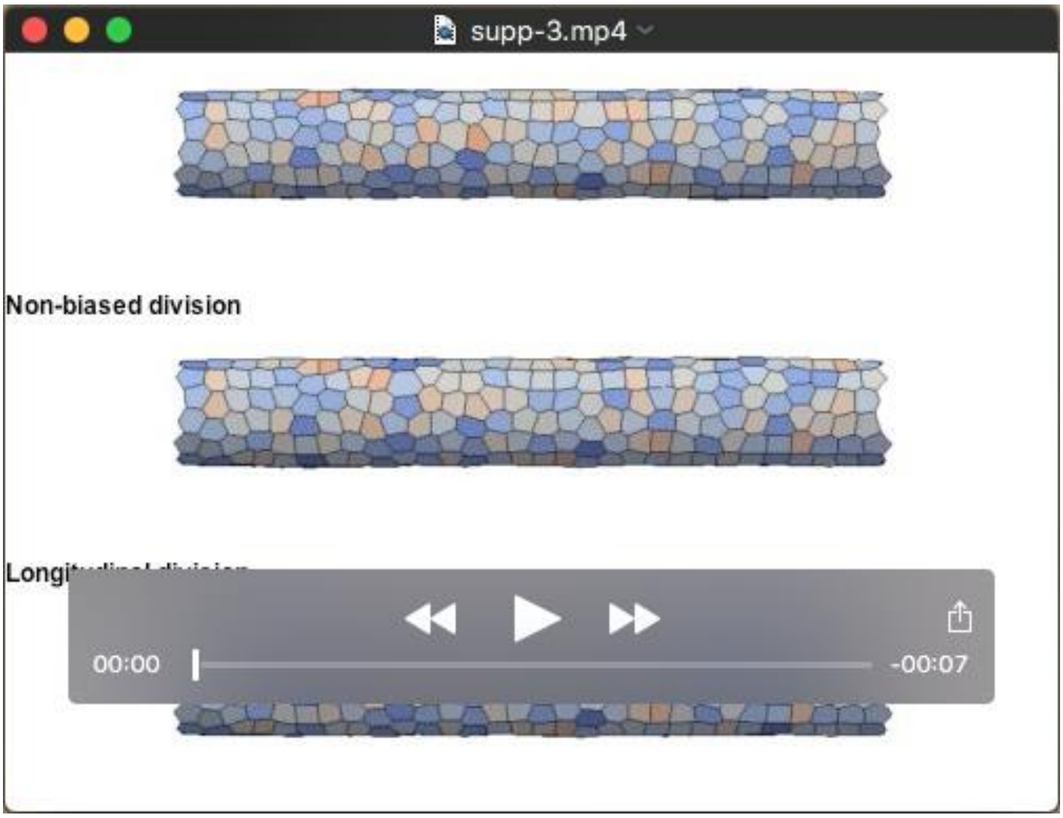

Movie 2. Model simulation in different cell division orientation, corresponding to the Figure 6

Development • Supplementary information

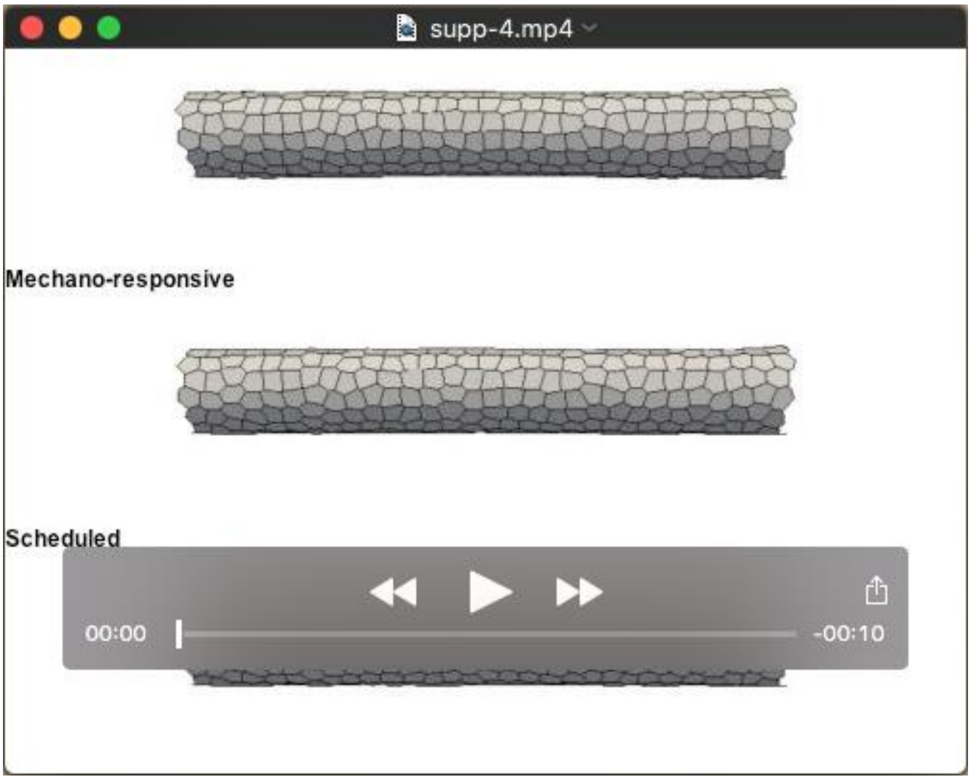

Movie 3. Model simulation in different regimes, corresponding to the Figure 6

Development • Supplementary information
